# Supplementary figures and images for: Correlated protein-RNA associations and a requirement for HNRNPU in the long-range recruitment of Polycomb Repressive Complexes by the lncRNAs Airn and Kcnq1ot1
Source: PLoS Genet. 2026 Jun 23;22(6):e1012215. doi: 10.1371/journal.pgen.1012215 (PMC13318007; doi:10.1371/journal.pgen.1012215)

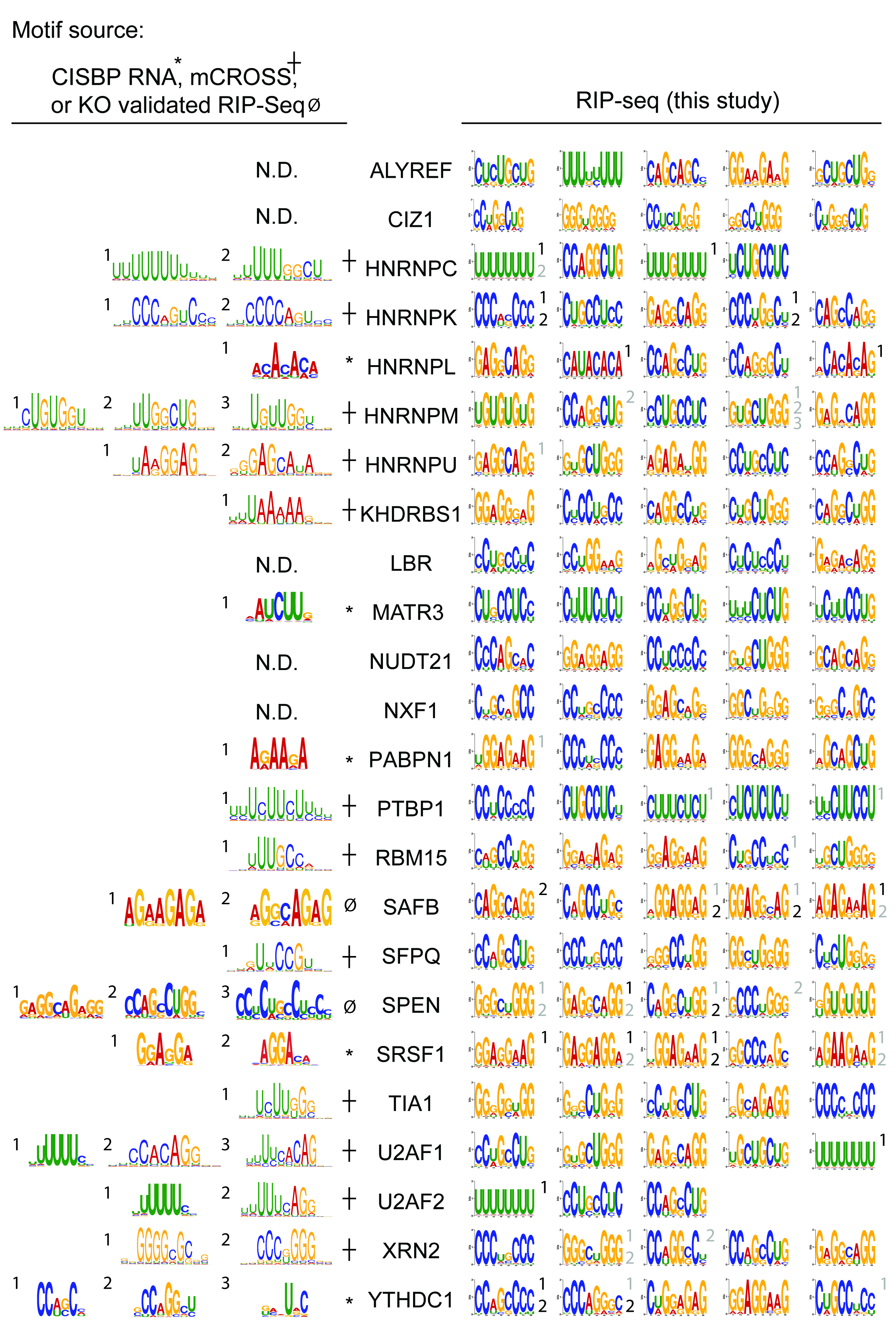

Supplement: S1 Fig — Left-hand panels, motifs reported in CISBP-RNA [139], mCROSS [140], YTHDC1 CLIP [93], or in the case of SAFB and SPEN, motifs derived from studies which used the same antibodies or antisera in this study and derived motifs by comparing RIP-seq from wild-type cells versus cells in which the denoted protein was knocked out [57,58]. Prior CLIP-seq performed to map the RNA targets bound by ALYREF, NXF1, and NUDT21 (a.k.a. CPSF5/CFIm25) failed to identify strong consensus motifs [141,142]. Right-hand panels, up to the top 5 motifs derived from RIP-seq experiments in this study. Tomtom was used to compare previously identified motifs to those identified in this study [124]; motifs on the right are marked by the number of the motif on the left with which they share significant similarity. Numbers on the right that are colored in grey signify a p-value of motif similarity of <0.05, and numbers colored in black signify a q-value of <0.05. The p-value of similarity between MATR3 CisBP-RNA motif #1 and RIP motif #5 was 0.057. RING1B, RYBP, and SUPT16H were not included in these analyses. (TIF) [file pgen.1012215.s001.tif]

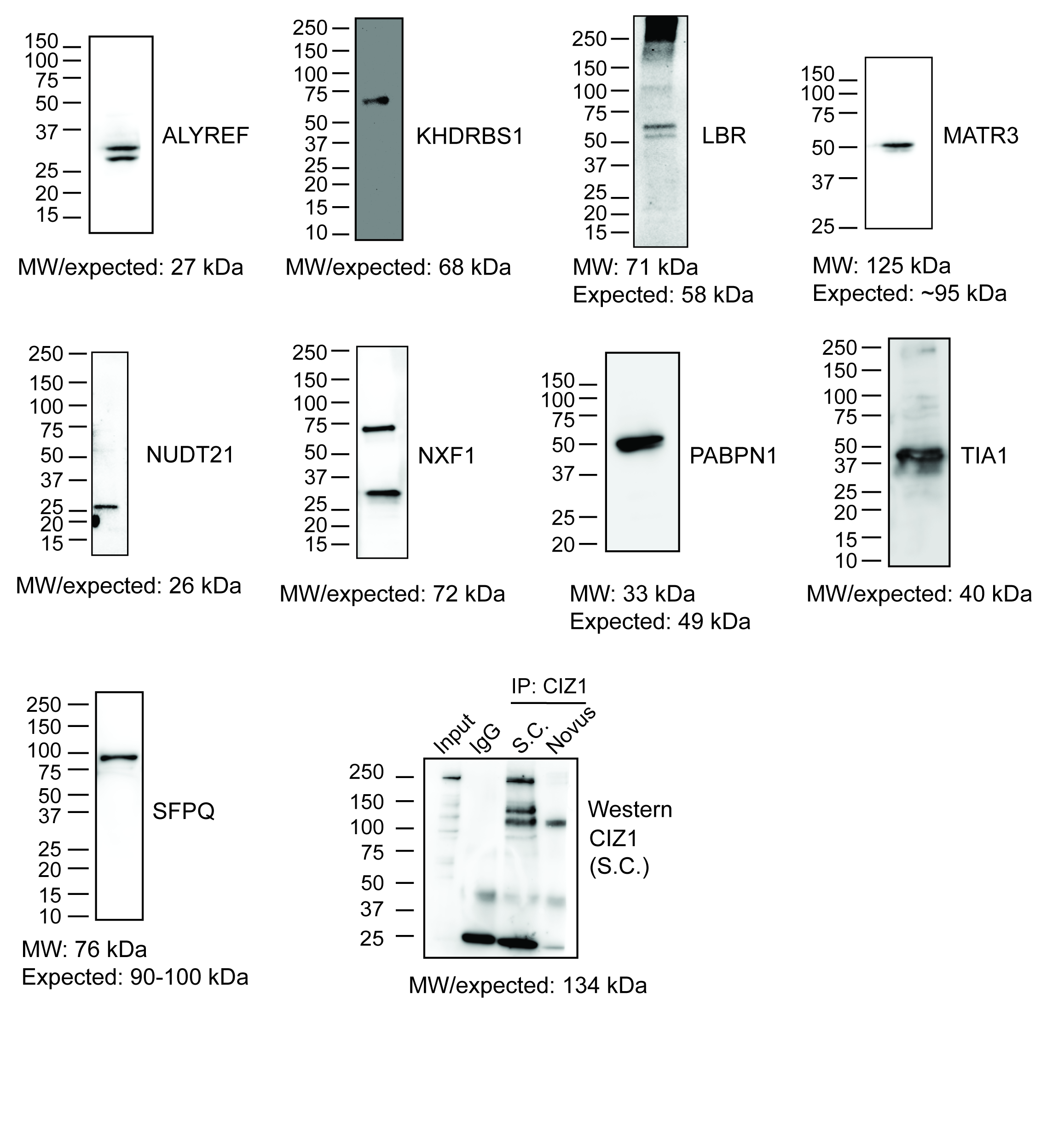

Supplement: S2 Fig — CIZ1 was only detectable by IP-Western; we used the CIZ1 antibody from Novus for RIP-seq in this study. S.C., CIZ1 antibody from Santa Cruz Biotechnology (sc-393021). IP western was performing using RIP-seq washes as described. (TIF) [file pgen.1012215.s002.tif]

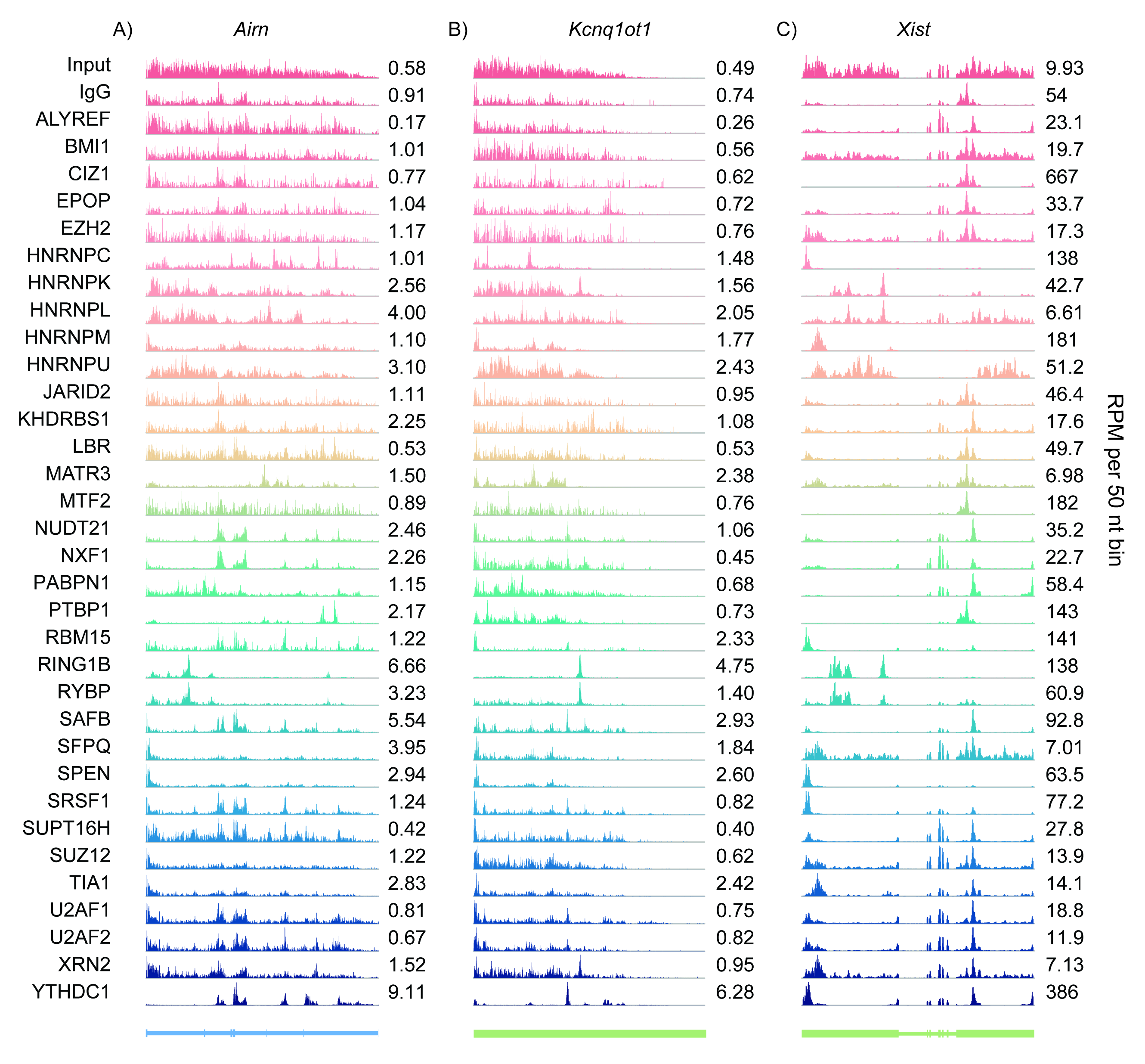

Supplement: S3 Fig — (TIF) [file pgen.1012215.s003.tif]

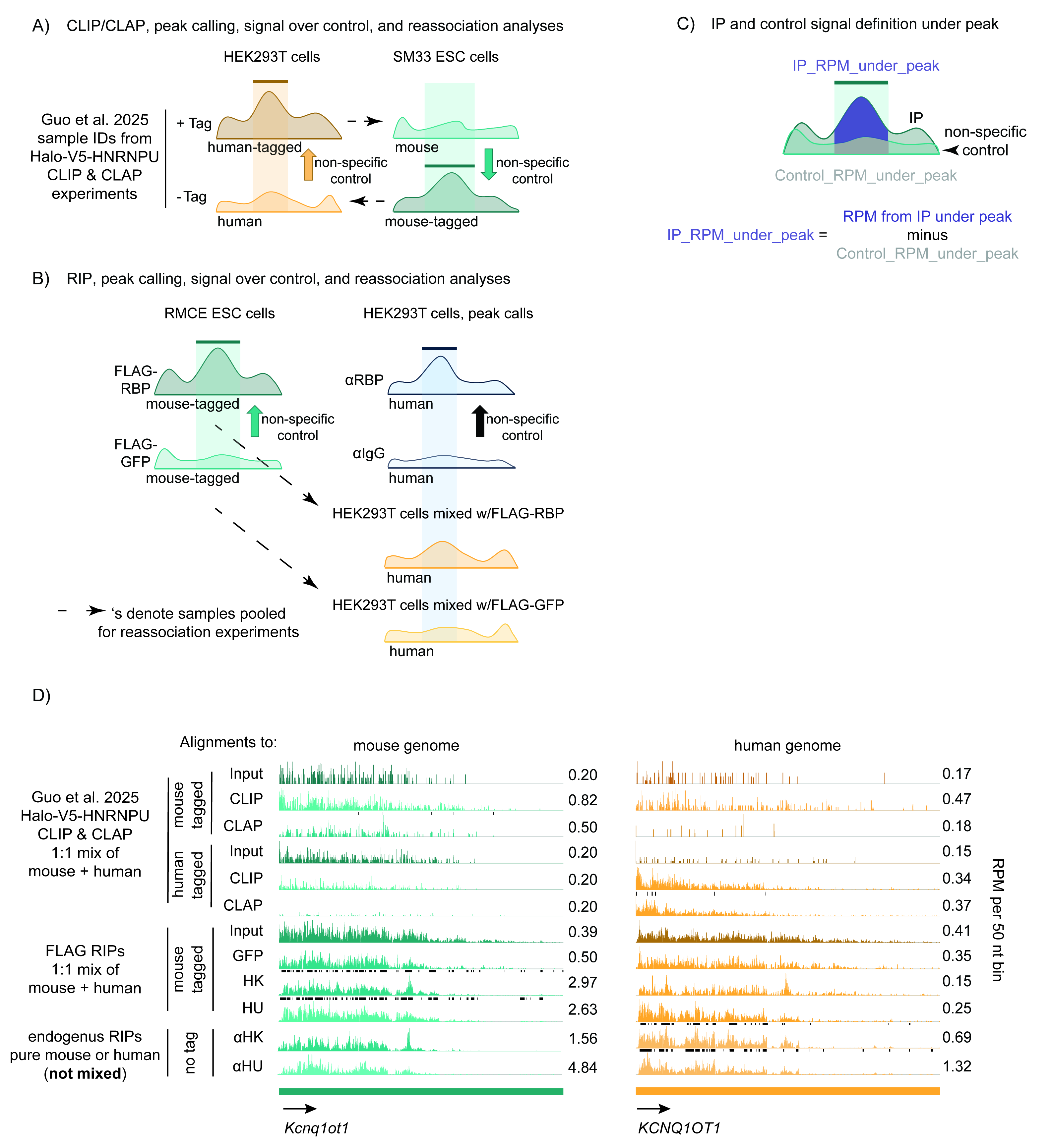

Supplement: S4 Fig — (A,B) Schematics demonstrating peak calling, signal over control, and reassociation strategies for CLIP and CLAP data from Guo and colleagues (A) [37] versus RIP experiments performed in this study (B). In all CLIP and CLAP samples: human 293T and mouse ESCs were mixed in 1:1 ratios. Peaks (shown as thick horizontal bars drawn above enriched regions) were called by comparing signal from the tag-expressing and tag-lacking genomes of the same species. For example, human peaks are called by comparing +Tag samples (which express the tagged RBP in human cells) to -Tag samples (which express the tagged RBP in mouse cells), while mouse peaks are called by comparing -Tag (which express the tagged RBP in mouse cells) samples to +Tag samples (which express the tagged RBP in human cells). Signal was assigned from the tag-expressing genome and non-specific signal was assigned from the tag-lacking genome of the same species. Reassociation analyses were conducted by calculating RPM signal under each peak in the tag-expressing species counting unique alignments to the tag-expressing genome, and separately, calculated RPM signal under each peak in the tag-lacking species using alignments to the tag-lacking genome and counting unique alignments to the tag-lacking genome. For example, in the mouse Xist HNRNPU CLAP reassociation analysis in Fig 1H, CLAP RPM signal under mouse peaks from the mouse-tagged-expressing ESCs (-Tag, third row, lefthand side of Fig 1I) was compared to CLAP RPM signal under human peaks in the tag-lacking human 239T cells that were mixed with the mouse-tagged-expressing ESCs (-Tag, third row, righthand side of Fig 1I). In FLAG RIP samples: FLAG-tag-expressing mouse ESCs were mixed with tag-lacking human 293T cells in 1:1 ratios. Peaks in mouse were called by comparing signal from the FLAG-RBP and FLAG-GFP samples. Peaks in human were called by comparing signal from RBP RIPs using the antibodies raised against endogenous RBPs versus IgG control. Reassociation an [file pgen.1012215.s004.tif]

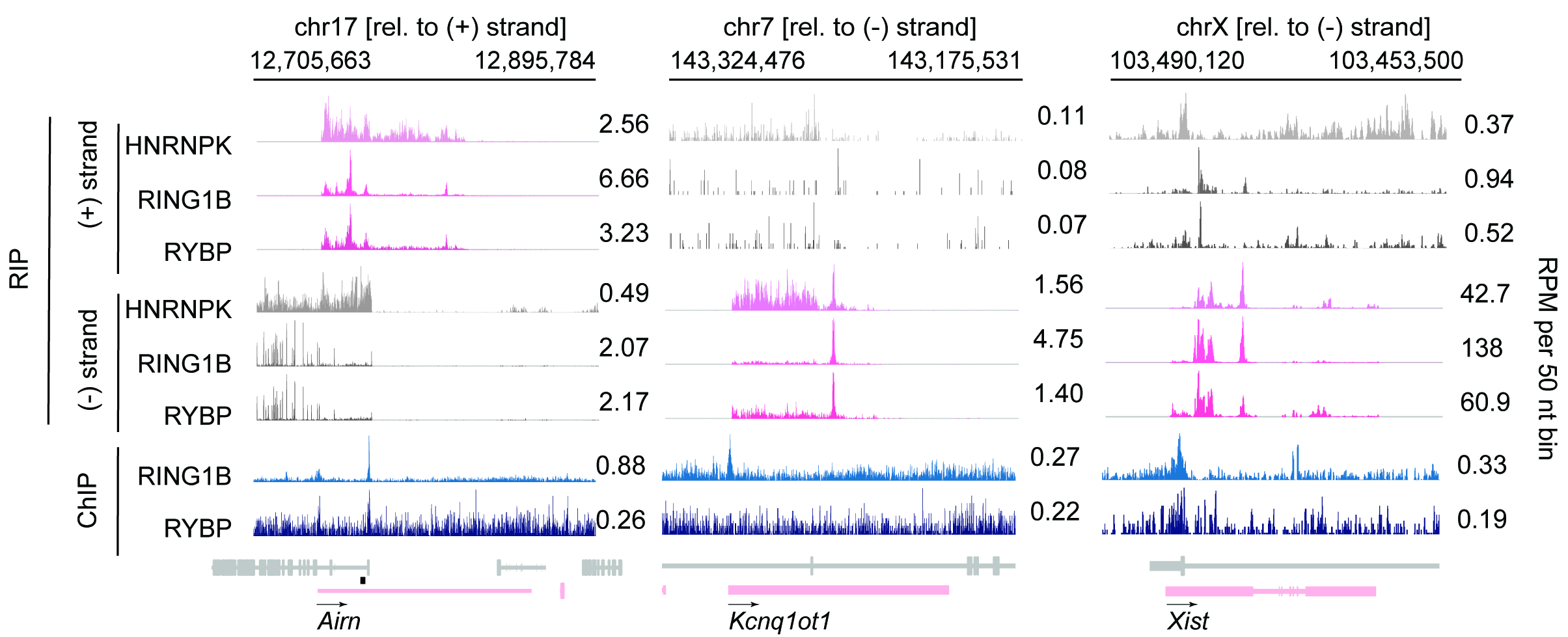

Supplement: S5 Fig — Wiggle density profiles of RIP, and ChIP data over Xist, Airn, and Kcnq1ot1 regions. Strand of interest (-) or (+) for RIP-seq and lncRNA in pink, antisense strand in grey. RPM, reads per million uniquely aligned reads per 50nt bin. Black bar above the Airn gene diagram marks the location of a co-localized RIP-seq peak for RING1B and RYBP whose genomic coordinates are proximal to but not overlapping in location to the ChIP peak for the same two proteins in TSCs. Maximum RPM values displayed in different rows were set to enable visualization of relevant trends. Intensity can be compared across rows as a way to gauge the relative levels of enrichment for each factor over the genomic intervals being displayed. (TIF) [file pgen.1012215.s005.tif]

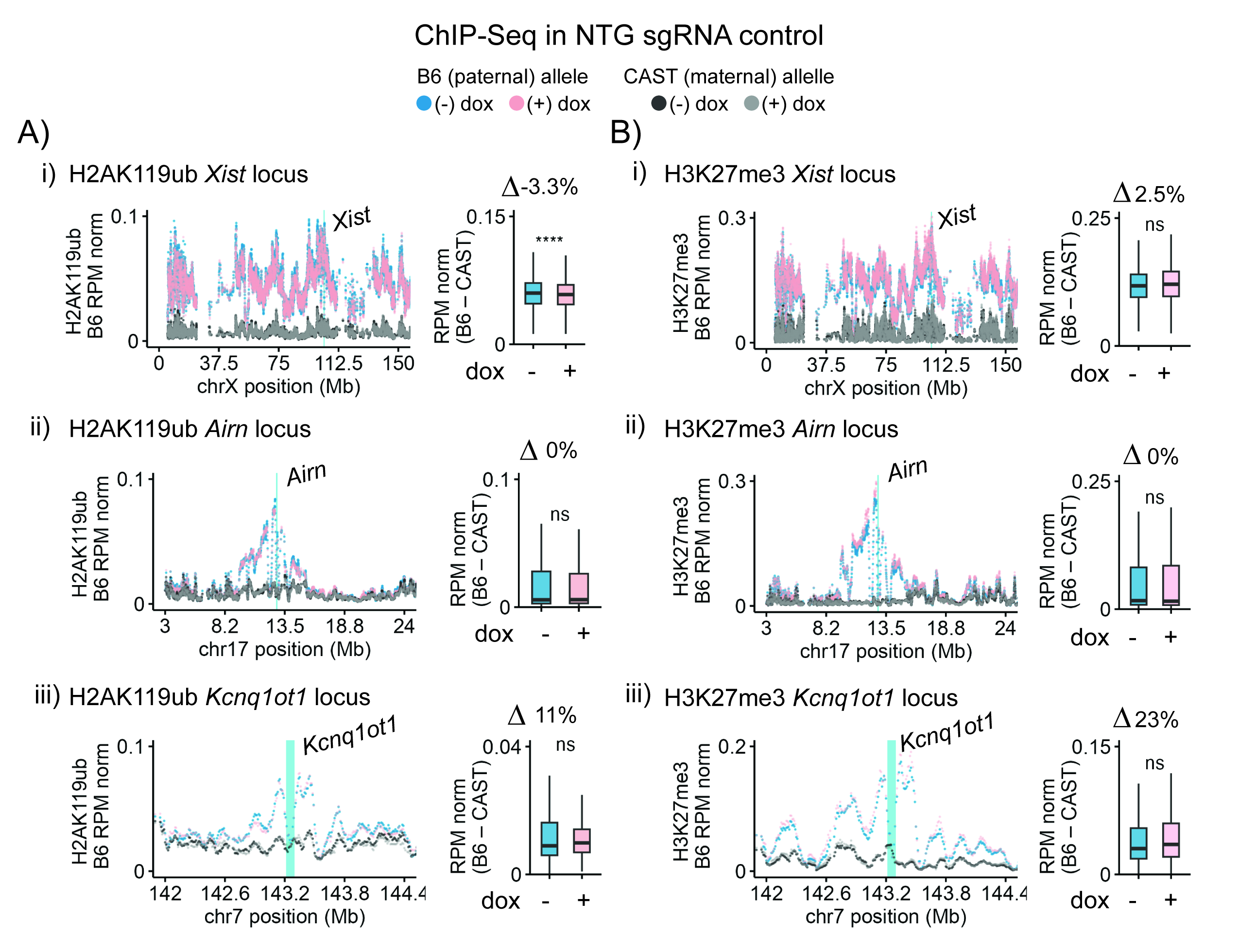

Supplement: S6 Fig — RPM-normalized H2AK119ub (A) and H3K27me3 (B) ChIP-seq data from TSCs expressing non-targeting sgRNAs shown over Xist, Airn, and Kcnq1ot1 loci. H2AK119ub and H3K27me3 levels are shown in panels (i, iii, v) and (ii, iv, vi), respectively, over the Xist (i, ii), Airn (iii, iv), and Kcnq1ot1 (v, vi) target domains, on B6 and CAST alleles with and without Cas9 induction by doxycycline treatment. Tiling density plots and box-and-whisker plots of spike-in normalized H2AK119ub and H3K27me3 ChIP-seq signal per 10 kb bin are displayed on the left and right, respectively, in both (A) and (B). Δ values above box plots show the percent fold change of median B6 minus CAST values between (-) dox and (+) dox within each lncRNA’s target domain. *, **, ****: p ≤ 0.05, 0.01, and 0.0001, respectively; Student’s t-test. (TIF) [file pgen.1012215.s006.tif]

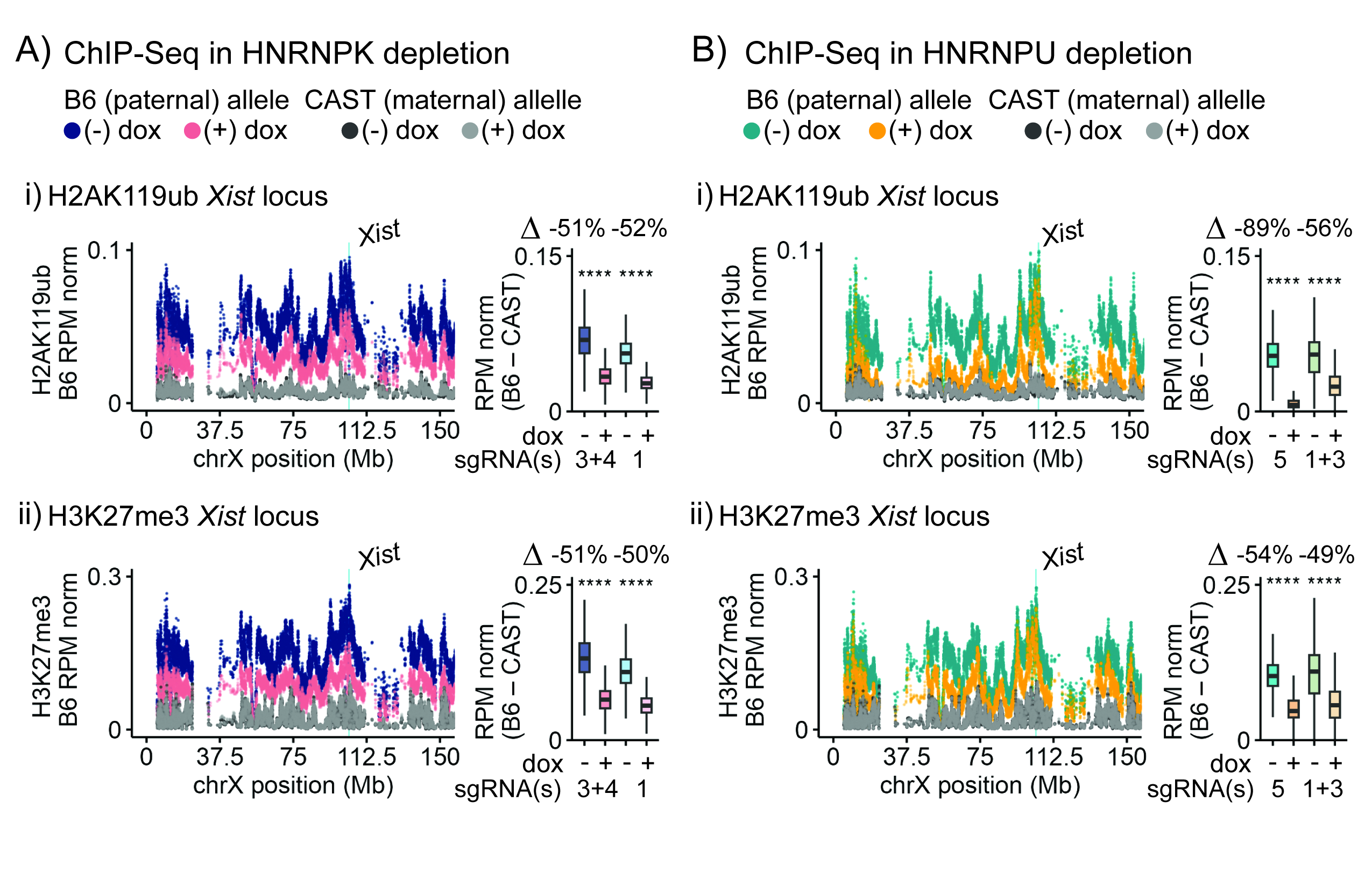

Supplement: S7 Fig — H2AK119ub and H3K27me3 ChIP-seq data in TSCs expressing HNRNPK-targeting (A) and HNRNPU-targeting (B) sgRNAs shown over Xist target domain. H2AK119ub and H3K27me3 levels are shown in panels (i) and (ii), respectively, on B6 and CAST alleles before and after four days of Cas9 induction with dox. Tiling density plots and box-and-whisker plots of spike-in normalized H2AK119ub and H3K27me3 ChIP-seq signal per 10 kb bin are displayed on the left and right, respectively, in (A-D). Tiling density plots represent the data averaged between the two different sgRNA-expressing populations, and box-and-whisker plots show [B6 - CAST] values for each sgRNA experiment. Δ values above box plots show the percent fold change of median B6 minus CAST values between (-) dox and (+) dox for each replicate over the X chromosome. *, **, ****: p ≤ 0.05, 0.01, and 0.0001, respectively; Student’s t-test. (TIF) [file pgen.1012215.s007.tif]

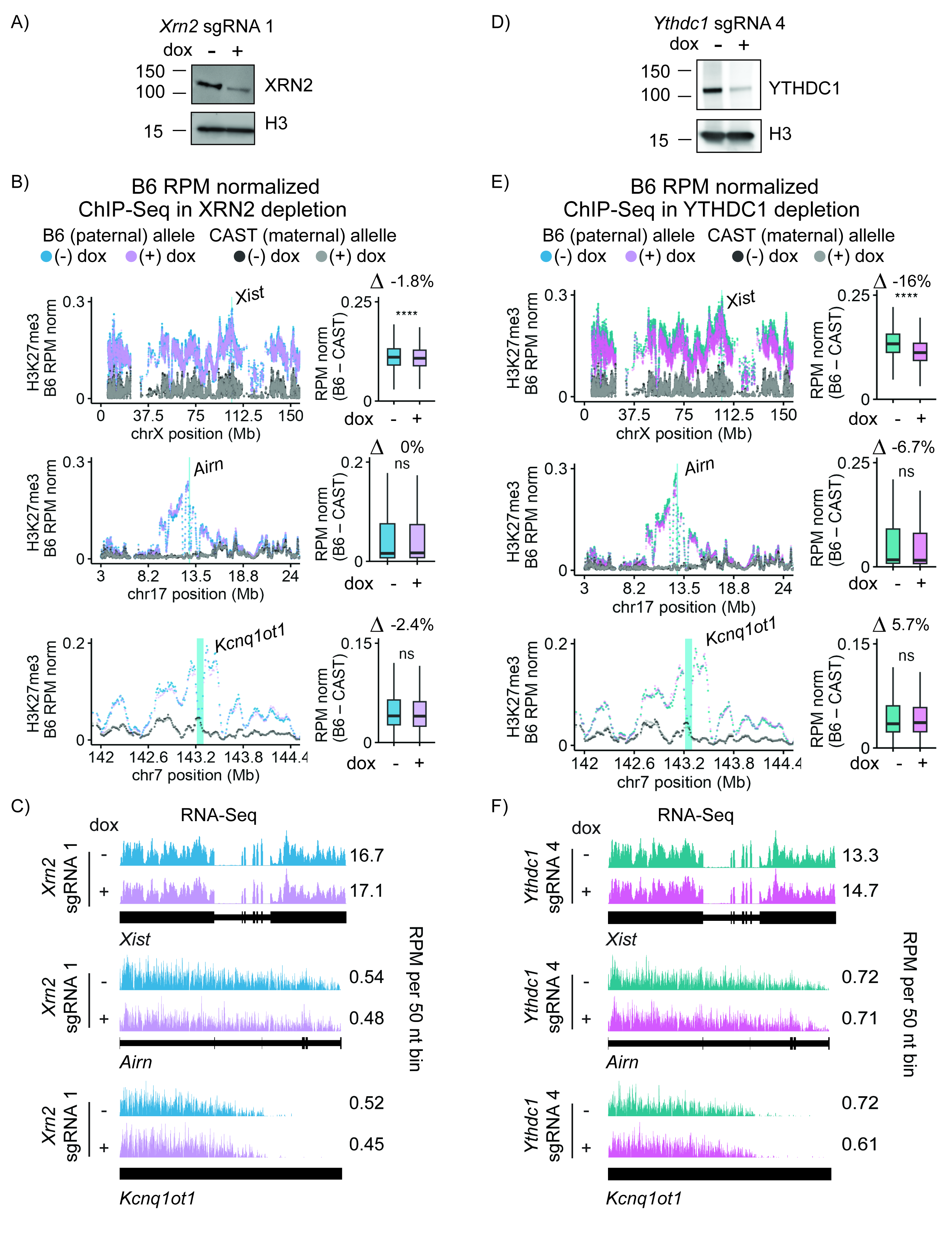

Supplement: S8 Fig — (A, D) Western blot of XRN2 and histone H3 in (-) and (+) dox conditions, in TSCs expressing Xrn2-targeting (A) and Ythdc1-targeting (D) sgRNAs. (B, E) RPM normalized H3K27me3 ChIP-seq data in TSCs expressing Xrn2-targeting (B) and Ythdc1-targeting (E) sgRNAs, shown over Xist, Airn, and Kcnq1ot1 target domains. H3K27me3 levels are shown on B6 and CAST alleles in (-) and (+) dox conditions (four days of Cas9 induction). Tiling density plots and box-and-whisker plots of RPM normalized H3K27me3 ChIP-seq signal per 10 kb bin are displayed on the left and right, respectively, in both (B) and (E). Δ values above box plots show the percent fold change of median B6 minus CAST values between (-) dox and (+) dox in each lncRNA’s target domain. *, **, ****: p ≤ 0.05, 0.01, and 0.0001, respectively; Student’s t-test. Data shown are from a single experiment. (C, F) Wiggle density plots showing RNA-seq data over Xist, Airn, and Kcnq1ot1 in (-) and (+) dox conditions in TSCs expressing Xrn2-targeting (C) and Ythdc1-targeting (F) sgRNAs. (TIF) [file pgen.1012215.s008.tif]

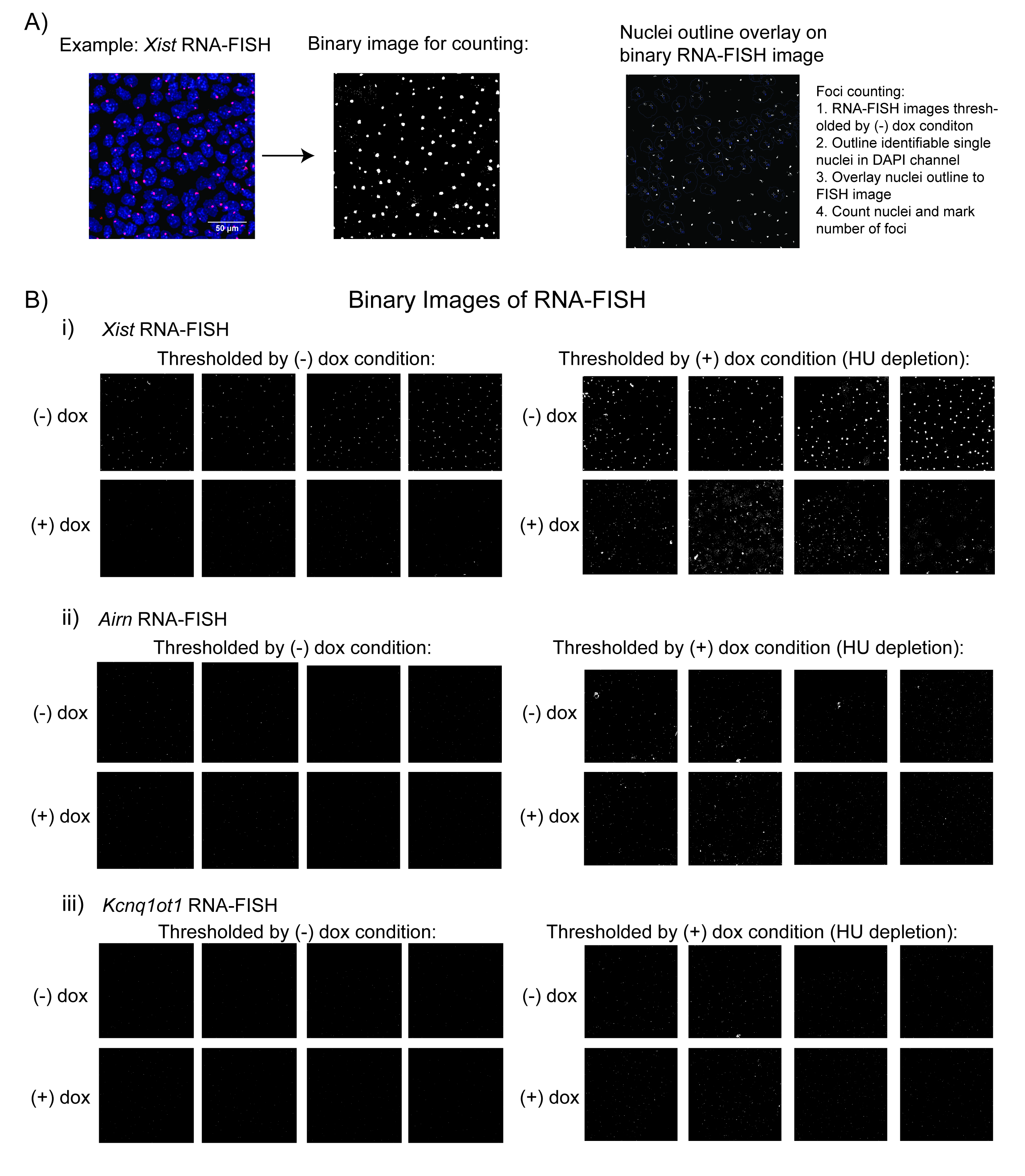

Supplement: S9 Fig — (A) Overview of foci counting strategy. (B) Representative Xist (i), Airn (ii), and Kcnq1ot1 (iii) RNA FISH images thresholded by signal in the (-) dox condition (left-hand panels) and (+) dox conditions (right-hand panels). (TIF) [file pgen.1012215.s009.tif]
