# Supplementary material for: Correlated protein-RNA associations and a requirement for HNRNPU in the long-range recruitment of Polycomb Repressive Complexes by the lncRNAs Airn and Kcnq1ot1
Source: PLoS Genet. 2026 Jun 23;22(6):e1012215. doi: 10.1371/journal.pgen.1012215 (PMC13318007; doi:10.1371/journal.pgen.1012215)
Supplement: Attachment — Submitted filename: response_to_reviews_round2_v1.docx [file pgen.1012215.s018.docx]

We would like to thank the editor and the reviewers for their continued evaluation and support. Reviewer 3 had a strong point and we have edited the discussion section and abstract to better highlight the effects that HNRNPU has on the abundance of *Airn* and *Xist*.

We have also removed a paragraph in the discussion section that we added in response to a first-round comment from Reviewer 2:

“While we did not perform direct comparisons between our protocol and native RIP or fRIP, we note that the wash conditions we employ are more stringent than those used in either protocol, and we might expect signal-to-non-specific-signal ratios to be higher as a result. Additionally, while developing our version of formaldehyde-based RIP, we compared different crosslinking procedures and found that the condition that we ultimately selected for use (30 minutes at 0.3% formaldehyde at 4°C) recovered higher signal over non-specific signal ratios in known regions of RNA-protein interactions relative to other crosslinking conditions, such as 1% or 0.1% formaldehyde for 10 minutes at room temperature. “

This particular paragraph did us a disservice by under-selling our process of protocol development, which occurred 10 years ago. In developing our protocol, I did directly compare it to native RIP and fRIP, and it outperformed substantially; hence, we moved forward with the protocol in the way that we did. However, I do not have publication quality data to report the comparison nor do I have the time or personnel to regenerate them – hence the statement that we did not perform direct comparisons, which was, in an unclear way, referring to the present work. The comparisons are unnecessary for the present study, which to my knowledge, already contains more substantive comparisons to previously published methods than any other study of RNA-protein interactions. Thus, the discussion section is cleaner and more accurately conveys the history of our work without the paragraph.

From Reviewer 3: Remaining major Point :

The authors did not address a main points raised by this reviewer (point 4):
In case there is indeed a change in the allelism of gene expression in the absence of HNRNPU, it remains unclear whether this is a direct or an indirect effect, and the authors need to be careful in their conclusions. For instance, in Figure 7c it can be seen that Airn lncRNA is less abundant in HNRNPU-deficient cells. This is unexpected given that they also observed a reduced presence of PRC-mediated histone modifications, and may well be an inditect effect of the HNRNPU depletion (the protein presumably controls many factors). Could this reduction in Airn lncRNA have been responsible for the observed minor change in gene allelism in Figure 5A? This seems a more plausible hypothesis. Further experiments, or considerable rewriting of the conclusions, would be required.

In the Discussion, the authors have to present and discuss the alternative model, namely that the minor allelic derepression of genes at the Airn-controlled domain in the HNRNPU deficient cells is an indirect consequence of the reduction in Airn levels-(of about 30%, Fig. 7c). A same reasoning could be applied to Xist, whose level is about 40% reduced in the HNRNPU-deficient cells. Given the importance of the dosage of these lncRNAs for their chromatin repressive effects, reported by the authors themselves and others in earlier studies, it is essential to indicate and discuss this plausible alternative hypothesis.

We agree – although we attempted to address this comment in our first resubmission, we recognize that we very much fell short. We have reworked the paragraphs in question to place the statements about HNRNPU’s effects on abundance first. We expanded the statements as well, and deleted a partially redundant and possibly confusing sentence at the end of the preceding paragraph. We edited the abstract to include a statement about the effects of HNRNPU depletion on *Xist* abundance, which we feel are important to highlight in this context as well.

Minor points :
-Figure 2A : Should the association of Malat1 to U2AF35 not be indicated in purple (because ranking =1) ?

Boxes in Figure 2A are shaded if by Wilcoxon rank sum test, the signal in our two replicates of RIP was deemed to be significantly higher than the signal in six replicates of non-specific IgG control. For U2AF35 and Malat1 the p value from Wilcoxon was 0.18 for the U2AF35 RIP, and thus did not meet our threshold for shading (Raw values reported in Table S2).

-Line 541 : FIG ?

This typo has been corrected; thank you for catching.
