# Supplementary material for: Correlated protein-RNA associations and a requirement for HNRNPU in the long-range recruitment of Polycomb Repressive Complexes by the lncRNAs Airn and Kcnq1ot1
Source: PLoS Genet. 2026 Jun 23;22(6):e1012215. doi: 10.1371/journal.pgen.1012215 (PMC13318007; doi:10.1371/journal.pgen.1012215)
Supplement: Attachment — Submitted filename: plos_G_reviews_v8.docx [file pgen.1012215.s017.docx]

We would like to thank the reviewers and editors for the time that they spent reviewing our work, for their constructive criticisms, and for their general enthusiasm for the study. We have addressed all comments and believe the manuscript has improved in clarity and impact as a result.

Reviewer #1: In their manuscript, Calabrese and colleagues examine similarities and differences in the networks of lncRNA-protein associations for three previously characterized lncRNAs (Xist, Airn, and Kcnq1ot1), and use these patterns to infer functional and mechanistic features.

The key significance of this manuscript is its focus on a network of 27 RNA binding proteins rather than on individual lncRNA-protein interactions. For this they use a new RIP (crosslinked RNA immunoprecipitation) protocol with validated performance and develop novel computational methods that identify patterns and develop predictive parameters for RNAs with similar/distinct protein association profiles. One of their key findings is that Airn and Kcnq1ot1, two lncRNAs involved in the repression of imprinted regions, have highly similar profiles that are also similar but to a lesser degree to Xist, another repressive lncRNA that acts on a chromosome-scale. Overall, both Xist and imprinting lncRNAs are significantly distinct from most other lncRNAs. The specific distinctions between Airn/Kcnq1ot1 and Xist and between the two imprinted lncRNAs are also highlighted as these might be representative of functional and mechanistic differences. Overall, the first part of the manuscript introduces a new framework for the comprehensive characterization of RNA/protein networks. This work extends prior work by this team that focused on lncRNA sequence kmers to identify functional similarities/differences.

The second part of the manuscript uses information from protein-association networks to select factors that are enriched for all three lncRNAs (hnRNP-K, -U, and Xrn2) or specific to the imprinting lncRNAs (Ythdc1) with predicted or known roles in gene repression. The authors achieve reasonable CRISPR inducible depletion for all 4 factors and perform allele-specific ChIPseq for Polycomb repressive marks (H2AK119Ub and H3K27me3) and gene expression analysis. They observe interesting similarities and differences in how hnRNP-U and hnRNP-K contribute to gene regulation by the three lncRNAs, which the authors interpret as analogous but partly distinct mechanisms of gene repression by the lncRNAs.

Overall, this manuscript lays the foundation for a new network-level approach for addressing the complex and combinatorial nature of lncRNA-protein interactions. The approaches are technically and computationally validated but may need more detailed descriptions in results and figure legends. The visualization of the data is excellent, but labels need to be updated for consistency and clarity. Lastly, jargon related to imprinting and X inactivation fields need more detailed explanations. Overall, improvements in the clarity of text and figures will make this manuscript accessible and of great interest to a wide audience.

We thank the reviewer for their time spent to provide such a thorough and thoughtful evaluation, for their constructive comments, and for their enthusiasm for the work.

(1) Explanation for the use of the specific model system. What are TSCs and why using these cells is appropriate for studying imprinting/X-inactivation?

We have now provided more background into our decision to use TSCs in this study (pages 6 and 7).

(2) It is unclear how do the authors define “chromatin-enriched RNAs”?

Thank you for pointing out this missing information, now stated in the methods and on page 12 of the manuscript. We defined a set of chromatin-enriched transcripts using a combination of total RNA-seq and fractionation RNA-seq previously performed in TSCs. Transcripts that were expressed above a median kallisto-defined expression threshold of 0.125 TPM (counts from total RNA-seq datasets), and whose ratio of [chromatin_RNA-seq_TPM]/([chromatin_RNA-seq_TPM]+[cytoplasmic_RNA-seq_TPM]) were greater than 0.75 (counts from fractionated RNA-seq datasets), were defined as chromatin-associated.

We also included total and fractionation RNA-seq data values for all analyzed transcripts in the new Table S3.

(3) Figure legends need improvements. In addition to obvious details such as definition of abbreviations (ie MoR and ERCC), panel content should contain descriptions accessible to a wider readership as currently they are too brief and schematic. There should be consistency across figures (ie sgRNA name/number/gene in Fig. 4, 5, and 6 or -/+ dox vs WT/HNRNPU in Fig. 6). Deviations from standard practice should be addressed (ie scale of Read density profiles is usually normalized to allow comparison of peak heights). Fig. 3B there is inconsistency whether 25- or 50-nt bins were used.

Thank you for this feedback. We have expanded our definitions of MoR and ERCC values in the legends to match those that are stated in the text of the main manuscript. We have corrected the misnomer "gRNA" used in Figures 5 and 6 to read "sgRNA" to be consistent with Figure 4 and standard nomenclature in the field. We have specified that the RNA-seq reported in Figure 5 is the result of merged biological replicates from both sets of sgRNAs reported in Figure 4.

Regarding read density profiles, we are following what is in our opinion a standard practice in the field, although Reviewer 2 also had questions about our axis labels so perhaps it is less standard than we think. In any case, we set max RPM values to be different in different rows to enable the visualization of relevant trends. Different antibodies exhibit different levels of enrichment and different levels of signal over non-specific signal. Plotting with variable axes is therefore necessary to display relevant trends. Because the data are RPM normalized, intensity can be compared across rows as a way to gauge the enrichment for each factor over the genomic intervals being displayed relative to the rest of the genome, but attention must be paid to the differences in scale of the y-axes. This information is now explicitly stated in the legend to Figure 1.

Note that while networks were defined using 25 nucleotide bins, the values in the wiggle tracks of Figure 3B are summarizing data in 50 nucleotide bins. There is no practical difference between 25 or 50 nucleotide bins; the two are essentially interchangeable in terms of trends detected. Raw RPM values for all RIPs performed can be seen in Figure S3.

(4) Related to (3), the text in Results section is often hard to follow, especially when referring to the methods development section. This is, in part, due to lack of consistent terminology. As one of several examples, Fig 2A is described as representing “summed background-corrected peaks across length,” “overall levels of association,” “RIP signal-under-peak rankings” and “signal-under-peak ranks.” This can be remedied by defining one metric and using it consistently.

Thank you for this feedback. Reviewers 2 and 3 also expressed a desire that we clarify terms. We have gone through the manuscript and attempted to clarify ambiguities and be more consistent with our use of terms. If we missed anything, we would be happy to correct it.

(5) Fig. 2C and 3C should also plot the ranking for a few other known lncRNAs that might be of interest readers (such as Malat1, Neat1, Hotair, etc).

Reviewer #2 also expressed a related interest. We have now included rankings for *Neat1*, *Malat1*, *Pvt1*, *Meg3*, and *Tsix* in Figures 2 and 3 and describe key differences and similarities among the lncRNAs in the manuscript (pages 13 and 14). We were happy to receive these suggestions and believe the comparisons help to draw out the exceptional features of Airn and Kcnq1ot1 and provide an additional measure of confidence in the approaches we used to map RNA-protein associations.

(6) There is a disconnect between order of panels in Figs 4 and S6-8 and related text, which makes this section very challenging to understand. The justification for selection of these 4 factors for further analysis based on relationship to PRC1 in Fig. 3A is also not clear.

We thank the reviewer for expressing these concerns. We have rearranged the section in way that we believe is more intuitive as well as removed a typo in which a supplemental figure was referenced prior to when it should have been referenced. During our revision efforts, we elected to remove our analyses of spike-in normalized data and now report only RPM-normalized ChIP data. This was in part a simplifying procedure that we think better reflects the reality of the experiments we performed; we did not perform spike-in normalization across all genotypes (namely, ChIPs from XRN2- and YTHDC1-depleted TSCs did not contain spiked-in human chromatin). Of equal importance, in ongoing experiments, we recognized that our normalization procedure was flawed in the sense that it relies on protein concentration in the lysate as a proxy to standardize amounts of chromatin added across samples, which we have observed is variable between replicates in a way that we believe is related to toxicity of protein knockdown/depletion. We are reluctant to report spike-in normalized values in this manuscript for that reason and believe that standard allelic RPM normalization and comparison between CAST and B6 alleles is a more robust procedure for our purposes. ChIP experiments in F1-hybrid cells are internally controlled even without external spike-ins because it is the difference in signal between B6 and CAST alleles within the same cell type that is being evaluated. Indeed, comparing our RPM normalized data to our prior analyses of HNRNPK depletion TSCs (PMID 31256989; spike-in normalized using Drosophila DNA), we see the same trends in the Airn, Kcnq1ot1, and Xist target regions. Our ChIP and RNA-seq data are likewise concordant; the relative reductions in H2K119ub and H3K27me3 that we detect on on the B6 alleles following depletion of HNRNPK and HNRNPU both coincide with low-level upregulation of genes from the B6 alleles in the Airn and Xist target domains, yet not the Kcnq1ot1 domain, which has the weakest chromatin phenotypes. Regarding the relationship to PRC1, we have added statements in the manuscript that we hope more clearly connect our line of reasoning to the experiments that we performed (see pages 19 and 20).

(7) Jargon in text and figures need to be updated to be more accessible to wider readership – for example when describing maternal/paternal alleles for imprinted loci or discussing degrees of X-inactivation escapees.

Thank you for this feedback. We expanded the legends and added additional statements and organizational changes throughout. We believe that the revised manuscript should be sufficiently accessible to a broad audience, but we are open to additional edits if discussions remain unclear.

(8) Gene expression representation in Figure 5 is challenging to understand. “% paternal” or “% paternal expression bias” needs to be clarified. Best if represented in different colors (B6/Cast -/+dox) across the locus, analogous to Fig. 4. The use of t-test to look across different genes does not seem justified as each gene may be regulated by a distinct mechanism.

Reviewer #3 also had suggestions for our allelic analyses in Figure 5, and we agree that we could have done more to describe the expression data. We also agree that at a granular level, individual genes likely have different dependencies that dictate their levels of escape and repression within lncRNA target domains. One commonality among the genes that are repressed in all three lncRNA-target domains is that their repression requires continued expression of the lncRNA; this is certainly true in the Airn and Kcnq1ot1 target domains because the genes we selected for analysis here have all been shown to exhibit repression that is dependent on continued expression of Airn and Kcnq1ot1 in TSCs (PMID 31256989); it is also likely true that many genes along the inactive X in TSCs require continued expression of Xist to maintain stable repression, although the data here are not definitive, possibly for technical reasons related to the toxicity of culturing Xist null female TSCs (PMIDs 16415857, 28468635).

This being said, we have added to Figure 5 and its accompanying text by including a gene-level analysis of significant changes induced by depletion of HNRNPK and HNRNPU, using an analysis procedure similar to the one we performed to study gene expression after Airn and Kcnq1ot1 truncation in 2019 (PMID 31256989). Consistent with the trends described in our domain-level analyses, we observe many genes in the Airn and Xist target domains whose overall levels of paternal expression increase modestly but significantly upon depletion of HNRNPK and HNRNPU. Perhaps consistent with the reviewer’s point above, the sets of genes whose expression change upon depletion of HNRNPK and HNRNPU are not entirely overlapping; although the levels of derepression are generally low, on the order of 4-8 percent, and we are underpowered to make informed statements about the possibility of gene-specific mechanisms of repression (pages 22 and 23).

We do believe a domain-level comparison of paternal expression ratios is justified and note that the overall changes we observe in paternal gene expression ratios are entirely concordant with the changes we observe in histone modifications; after depletion of HNRNPK and HNRNPU, we see stronger reductions in PRC-directed chromatin modifications in the Airn and Xist target domains than in the Kcnq1ot1 target domain, and when we compare paternal gene expression ratios from RNA-seq , the exact same trends are observed. We agree that t-test is not quite appropriate here and have substituted that test with the non-parametric Wilcoxon rank sum.

(9) In Fig. 7, location of qPCR primers should be indicated.

We have included the location of qPCR primers in Figure 7.

Reviewer #2: The manuscript entitled "Correlated protein-RNA associations and a requirement for HNRNPU in long-range Polycomb recruitment by the lncRNAs Airn and Kcnq1ot" by Murvin et al. presents a formaldehyde crosslink based method for RNA protein interactions and applies it to investigate the binding of 25 RNA binding factors and 2 Polycomb group proteins to transcripts in B6/CAST hydrid mouse tophectodermal stem cell cultures. The use of hybrid cells allows the allele specific assignments of interactions which is important for detailed analysis of the interactome of the imprinted and non-protein-coding Airn and Kcnq1ot1 RNAs. In addition, crosslinking allows to capture indirect associated proteins, which is used to determine the dependence of interactions on the RNA binding protein hnRNPU, which is identified as an interactor of Airn, Kcnq1ot1, and Xist.
Comparison between the 3 lncRNAs based on extended analysis using available databases of protein interactions indicates similarities and differences, whose functional significance remains untested. The authors further observe a role for HNRNPU for establishment of the Polycomb complex associated histone modifications H3K27ME3 and H2AUB by performing sgRNA mediated depletion. This is an interesting observation as hnRNPU has not been directly implicated in Polycomb recruitment before, but in the localization of Xist RNA to the inactive X chromosome. A similar conclusion for AIRN and KCNQ1OT1 cannot be made suggesting potentially divergent requirements for hnRNPU between different non-coding RNAs.
The study can make an advance on the methodology for RNA-protein interactions, although the present version falls short in clearly positioning the new method over earlier publications using crosslinking and antibody mediated enrichment strategies. The biological insight gained is limited but might be a valuable resource for protein binding in mouse trophoblast stem cells, which could be of interest for researchers in RNA biology and stem cells. Before publication can be considered a number of points should be addressed by the authors to improve the clarity and strengthen the conclusions of the study:

We thank the Reviewer for their time, and for their thorough and thoughtful review. We respond to specific comments below.

Specific points

1. The authors use 6 replicates with non-targeting IgG to control for specificity. However, this does not rule out any cross-reactivity of the actual antisera used for the different interactors. This is a critical issue as the interpretation and usefulness of the binding data as a resource depends on. Therefore, controls for antibody specificity in addition to IgG are needed. The data in Fig. S2 Western blot raises this concern directly as multiple bands for ALYREF, LBR, TIA1, and NXF1 are observed. In addition, the antisera for detecting CIZ is stated to only yield specific bands by IP WB, which in the figure panel also does not appear as a single clear band. The image for MATR3 MW seems to indicate 125 and 95 KDa expected, but the band seems to be closer to the number 50, which makes it unclear how to interpret this critical control.

We agree that non-specific IgG has limitations as a control and direct the reviewer to the summary of our perspective stated in the results section, which we feel echoes the concerns that they expressed here (page 7). See also the text in the "Limitations of the study" section (page 31). We also agree with this reviewer that we under-explained the western blot data in our original submission and have edited the text accordingly (page 8).

Regarding the request for additional controls, we note that for the proteins that we focused on in the second part of the manuscript ( HNRNPK and HNRNPU), we include genetic perturbations and epitope-tagging experiments, both of which accord with the conclusions drawn from the monoclonal antibodies used for RIP in TSCs. For the remaining RIP datasets, we and others in the field who may be analyzing these data in the future will need to heed the concerns implied by the reviewer and which we articulated in our discussion section.

2. The conclusion at the end of the section on page 12 "Thus, the protein association profiles measured over Airn and Kcnq1ot1 are more similar to each other than to the vast majority of other chromatin-enriched RNAs and exhibit a more moderate degree of similarity to Xist." raises the question if the differences would be explained alone by the greater abundance of Xist relative to Airn/Kcnq1ot1. It could also be considered that the half-life of these RNAs when let us say the RNA binding proteins were grouped by their presumed on rates. As the section seems to contain a large number of statistical detail including rank numbers, the overall positioning of what this can tell us should be improved to make a convincing test case for the usefulness of the method.

Recognizing that we may have a different opinion than the reviewer, we believe that our conclusions in this section were appropriately measured and justified. Still, the suggestion to compare expression to protein-binding patterns was well received and we performed the analyses suggested. There is no correlation between expression and protein association profiles, although there are low but significant correlations with length. We note that Airn and Kcnq1ot1 are exceptionally long and unspliced RNAs, and evidence would suggest that their length is one of their core features that enable them to coordinate long-range gene regulation; thus the correlation between length and protein associations likely reflects a biological reality, in our opinion. Please see page 15.

2. In Figure 3 the peaks for Ciz1 and PTBP1 seem to overlap with peaks at the same positions the in IgG control. Maybe I have misunderstood the figure, but an explanation is needed as if indeed the peaks would be observed in the IgG control how could this experiment be interpreted?

The height of the peaks provide insight into the Reviewer's question. In contrast to a typical ChIP experiment, non-specific association in RNA-based enrichment approaches can be highly non-uniform. Still, in regions that experience high levels of non-specific association, specific RNA-protein associations can occur. Within such regions, interpretation of specific signal becomes more challenging, and orthogonal validation is needed before high-confidence interpretations can be made.

In the RBP data plotted in Figure 3B, each 50nt bin in the RIP tracks has had the corresponding RPM density from the IgG control subtracted from it. If one takes the maximum value of RPM in the grey non-specific IgG track at the top of the figure panel, and adds that value to the maximum value in the Ciz1 and PTBP1 tracks, it can be inferred that on an RPM-normalized basis, the signal in the PTBP1 and Ciz1 tracks are both >2x higher than non-specific IgG control in their main enriched regions. Raw RPM normalized RIP signal for all proteins profiled can be observed in Figure S3.

In the instance being described by the reviewer, the maximum enriched region in *Xist* occurs over Repeat E, a region that is known to be a bona fide PTBP1 and CIZ1 interacting region. Were the focus of our manuscript to be Ciz1 or Ptbp1, we would have pursued additional experimentation to understand the significance of potential enrichment. Although we make no specific statements about Ciz1 and Ptbp1 in our manuscript, in this instance, the biological validation has already been performed (PMIDs 28546514, 28923964, 32908311). Should the reviewer wish to read about it, another example of bona fide RBP interactions being detected in regions that also accumulate high levels of non-specific signal can be seen in a manuscript that we have posted in the bioRxiv, in which we found that SRSF1 is a *Xist*-Repeat A-binding protein important for SPEN recruitment by *Xist* (PMID: 40502125). Like Repeat E of *Xist*, Repeat A of *Xist* is associated with high non-specific signal; yet, it is among the most important RNA-protein interaction domains in *Xist* and within it, specific RNA-protein interactions occur and can be detected by our version of RIP (and other methods such as CLIP and CLAP).

In an attempt to increase clarity, we have re-labeled the y-axes of the Figure 3B plots to be (RIP_RPM - IgG_RPM).

Raw RPM values across Airn, Kcnq1ot1, and Xist for all RIPs performed can be seen in Figure S3.

3. I am wondering if the sharp peaks which the authors observe for Rybp and Ring1b might be caused by DNA contamination? In this case it is conceivable that the proteins are binding chromatin independent of RNA and a clarification to what extent contamination with chromatin DNA can be ruled out should be added.

This was an insightful comment. When developing this protocol in 2017, we included steps that effectively eliminate DNA contamination. Minimal sonication leaves genomic DNA fragment size too large to be amplified in library purification protocols, the use of Trizol (acidic phenol) causes the majority of DNA to partition to the interphase, and the use of an on-column DNA digestion step removes most if not all remaining DNA contamination. Minus RT controls in qPCR-based assays serve as a conservative estimate of DNA contamination because they can amplify long genomic DNA that would not be expected amplify in library preparation procedures. Still, in our RIPs, minus RT controls routinely amplify nothing (see PMID 41455104 for an example), supporting the success of the steps included in our protocol to eliminate DNA contamination.

This being said, in a 2023 study, we accumulated ChIP data for RING1B and RYBP within the same TSC line that was used for RIP in the present work. Comparing alignments of our RIP data relative to our ChIP data demonstrate that as expected given the robust elimination of DNA contamination in our RIP procedure, there are no overlaps between RIP and ChIP peaks. The reviewer can also observe that the RIP data are strand-specific, which requires that they originate from RNA and not DNA, the latter of which would have appeared as dual-stranded alignment under RIP-enriched regions (see page 13 and Figure S5).

4. Tsix overlaps Xist on the reverse strand and has also been implicated as a major repressive RNA. It would be interesting to know if the authors have detected Tsix in their FLAG-HNRNPK and FLAG-HNRNPU RIP discussed in the beginning of the results section. As the data is likely available, this could be an interesting case as Tsix is published as expressed in mouse ES cells and might be of a similar abundance as Airn or Kcnq1ot1.

It is true that *Tsix* is expressed in TSCs, although we do not consider *Tsix* in the same functional class as *Airn* and *Kcnq1ot1* because to our knowledge, it silences only one gene by antisense transcription and not via the long-range induction of PRC-directed chromatin modifications. Still we shared the Reviewer's interest and note that R1 had a similar request, and agree that these were worthwhile additions to the manuscript. We included *Tsix* in a small panel of other lncRNAs in our assessment of ranked signal as part of the revised Figure 2 and Figure 3.

5. Fig. S8 shows that the depletion of XRN and YTHDC1 is reducing but likely not completely eliminating the protein leaving the possibility open that these proteins might require a lower abundance for fulfilling their functions. Accordingly this limitation should be added to the text on page 17 line4 when concluding that both proteins are not essential.

We have included a statement describing that limitation at the end of the section in the manuscript that describes the data in Figure 4 (page 21).

6. On the lower part of page 20 the authors explain hat their protocol has been adapted from an earlier fRIP method which has been used by the authors before. On one hand this seems to serve to reassure that the method can provide high quality data, but on the other hand this statement also leaves the novelty of the method unclear. The positioning of the study would be much improved by clearly discussing the innovation in this manuscript against earlier work. This is important as the biological advances that can be made appear rather limited, and a clear advance on the methodology could strengthen the impact of the study.

We thank the reviewer for helping us to draw out this aspect of our study and want to be clear that the protocol in question is essentially the same one that we have been using in the lab for the last eight years (first versions published in PMIDs 30510238 and 31256989). In the time since Guttman and colleagues published their work examining RNA association with PRC2 and CTCF by CLAP (PMID 38387462), we have begun to encounter colleagues who confuse our version of RNA immunoprecipitation with a protocol published by Mili and Steitz >20 years ago (PMID 15388877), which, in contrast to our own protocol, did not crosslink samples, did not fragment RNA, and did not report washing beads post-immunoprecipitation. Perhaps not coincidentally, that same RIP protocol suffered from what appeared to be excessive levels of non-specific background (PMID 15388877). We do not claim novelty of our own protocol in this study; it is the benchmarking against CLIP and CLAP that is significant, arguably even more significant than the biological insights reported in Figures 2-7. It could be argued that even in the Guttman study, CLIP and CLAP were not benchmarked on equal terms or in an quantitative way. Without the benchmarking reported here, we and others in the field may continue to carry forth concerns about RIP and CLIP protocols that are largely misguided. CLAP certainly has strengths as a protocol, but in our analyses, it exhibits marginal improvements over RIP and CLIP yet comes with the cost and risk of having to epitope tag proteins of interest. Confusions between existing protocols can be harmful to progress, and in the worst case scenarios, they can cause manuscripts and grants to be rejected on mistaken grounds. Thus, we remain excited for the opportunity to publish our benchmarking experiments, not because of the novelty of the protocol, but because we feel an urgent need to report a direct comparison of our version of RIP to CLIP and CLAP; all three protocols offer their own strengths and should be used as needed by the scientific community.

In our original submission, we dedicated three paragraphs in the discussion section to highlight some version of these points. Based on the suggestion, in our revision, we attempted to more clearly highlight the importance of the benchmarking, as well as inserted additional comments that may help others understand the usefulness of our RIP procedure. Relative to native RIP and the “fRIP” protocol reported by the Rinn lab, we note that our version of RIP uses substantially more stringent washes and a different crosslinking procedure that in pilot tests, improved signal over non-specific signal ratios within positive control regions. We have now stated this in the discussion section. We continue to view the comparison between formaldehyde and UV-based crosslinking as a clear point of interest for the future (page 26).

Minor points

a) Pg 6: "non-FLAG antibodies" could be replaced by a more specific expression

We have made this change.

b) Pg 10: "CLAP generally although not always recovers less noise, whereas RIP can recover higher signal intensity under peak regions, presumably due to efficiency of formaldehyde crosslinking": I am wondering if the conclusion on page 10 could be rephrased to indicate specificity and non-specific binder as better quantified either by probabilistic modelling of absolute percentages. Noise and signal seem to be meaningful only in the computational domain but it is difficult to understand a molecular biochemical relation. It could be considered to replace noise, background and other synonyms with non specific binders throughout the text to easy understanding, unless their is a specific reason for the synonym which should be made clear to the reader.

Reviewer 1 also had suggested that we clarify terms and do more to keep them consistent. We agree that non-specific binding accurately reflects the intent and have changed its synonyms to “non-specific” throughout the manuscript.

c) Unspliced lncRNA have recently been proposed as being targets of the HUSH complex. The authors emphasize a role of Polycomb but does not consider HUSH contributions to gene silencing.Since all 3 RNAs have large first exons and likely limited splicing a reference to the literature and why HUSH dependence on HNRNPU or K has not been considered should be included in the discussion.

To our knowledge, prior epigenomic studies have found no obvious enrichment for H3K9me3 in the genomic intervals targeted for repression by *Airn*, *Kcnq1ot1*, and *Xist*; although all three lncRNAs do appear to engage enzymes that lead to the nearby deposition H3K9me2. Additionally, Almeida and Brockdorff have recently shown that HUSH represses *Xist*. In terms of lncRNA abundance, we see the opposite phenotype of Almeida and Brockdorff; HNRNPU and HNRNPK depletion decrease the abundance of *Airn* and *Xist*. We could and perhaps should be considering HUSH as a PRC1-like co-factor for these lncRNAs, but in this particular study, for better or worse, HUSH did not play a part. In the absence of any hard connection between HUSH and the experiments that we conducted, we did not feel justified in speculating that K or U direct HUSH to nearby regions in cis. However, it seemed reasonable to speculate that K or U serve to partially protect *Airn* and *Xist* from HUSH, and we did so in the revised discussion section – see page 30.

d) The references for the requirements of HNRNPK and SPEN for silencing should be separated on page 4 last sentence to make attribution clearer.

We have done so and edited the sentence to more accurately describe the findings that were reported by Quinodoz et al. As a reviewer-only comment, we recently observed that in our own SPEN knockout ESCs (not analyzed in the current study), *Kcnq1ot1* levels increase dramatically and coincide with increased silencing of several of its target genes; the opposite of what was reported by Quinodoz et al., who did not analyze Kcnq1ot1 target gene expression in SPEN knockout cells.

Reviewer #3: In this thorough, well presented study by the team of Mauro Calabrese, the authors explored which proteins could be associated with the regulatory lncRNAs Xist, Kcnq1ot1 and Airn in murine TSCs. As a basis, they took all the proteins known to be associated with Xist. Importantly, they compared a novel, adapted RIP protocol with existing CLIP and CLAP methodologies previously published by others. This allowed them to determine with a higher degree of certainty which proteins are associated with the three different lncRNAs. Interestingly, they find patterns of protein associations that are similar between Airn and Kcnq1ot1, and one of the new proteins identified was HNRNP-U. In a second part of the study, they performed functional studies on this protein and found that its association is important for the maintenance of histone methylation controlled by PRC1 and PRC2 complexes. In addition, they describe an effect of this protein on the allelic repression of target genes of Airn, but here the data are less convincing. Overall, this study provides novel insights into the gene repression controlled by the three lncRNA, and represents an important to the field. Nevertheless, this reviewer has several major concerns and about the data and their interpretation, and this is explained below.


We thank the reviewer for their time, for their enthusiasm, and for their constructive suggestions below.

Major concerns :

1) The conclusions are mostly based on analysis of a single mouse hybrid TSC line only. This line was derived many years ago (Calabrese et al. 2012). Since stem cells can readily deviate during in vitro culture, and upon manipulation, both epigenetically and genetically, it is important to ensure that this single female TSC line was still appropriate for the studoies at the analysed passages. In particular, the authors need to demonstrate that the allele-specific DNA methylation at the promoter regions of Airn and Kcnq1ot1 is unaltered in these female TSCs at the passages used (maternal allele only, 50% overall level). To be able to draw general conclusions, it is also important to demonstrate that this cell line had an unaltered karyotype.

We note that TSCs are difficult and expensive to culture and we do not maintain them in passage any more than is absolutely necessary. As a result, the difference in passage number between the TSCs used in our 2012 study and the present study is between 10-15 passages. We re-genotyped our lines in 2015, confirming their largely diploid nature prior to a study that we published in G3, but we no longer have access to those raw data and are unable to report them here.

However, we were able to examine allelic histone total histone H3 ChIP-seq signal across chrX, the Airn domain (chr17), and the Kcnq1ot1 domain (chr7) in datasets from our earlier studies (2011) and from total histone H3 ChIP-seqs performed in the present time (2026). In all datasets, B6 and CAST H3 signal are consistent among 2011 and 2026 samples, with no evidence of allelic imbalance that would indicate a change in karyotype at our loci of interest or genome-wide; see the example figure below. These data are also consistent with the allelic ChIP tracks shown in Figure 4, and with the allelic expression data now reported in Table S5, both of which confirm the relatively diploid nature of our TSC lines and their karyotypic stability which has been maintained since their derivation. We are not stating that the TSCs used here or in any other of our studies are perfectly diploid. For example, from the total H3 ChIP data as well as our genotyping in 2015, it is clear that the TSC line used here has a duplication of the B6 copy of chr11. However these abnormalities are not present in our regions of interest and appear to be stable over the timescales that we have been working with these cells. From the allelic expression data in particular, the reviewer can appreciate that our three lncRNAs of interest are expressed with the expected extreme levels of allelic biases, and that their target genes on chr17, chr7, and chX exhibit the opposite allelic biases, also as expected. The allelic numbers show no significant differences from when we first reported them in 2012, when we reported our second study of autosomal imprinted regions reported in 2015, or when we reported our third and fourth studies in 2019 and 2023. Based on the allelic biases, the DNA methylation profiles of the promoters of the lncRNAs can be inferred to remain as expected.

2) Ideally, however, the authors should complement their data with a selective experiments on an independent second TSC line, if possible of reciprocal genotype. Such lelective experiments on a second line could confirm their finding that HNRNPU influences levels of allele-specific histone modifications controlled by PRC complexes, and could strengthen the supposed effects on the levels and allele-specificity of expression of the Airn target genes.

We agree that knockdown of HNRNPU in a reciprocal line would provide additional insights and another measure of confidence that the results we observed in our C/B TSC line are generalizable beyond this single cell line. However, it is also true that genetic perturbations in TSCs are exceptionally challenging and as a lab we have far exceeded the amount of resources available to us for carrying out this work. It is not feasible for us to continue these studies at this time. We stress that our results from our C/B TSC line were replicated to a high standard; concordant results from two independent replicates using monoclonal antibodies raised against two separate histone modifications, along with concordant results from four biological replicates (16 total) RNA-seq experiments that show a mild derepression of genes on the paternal alleles after HNRNPK and HNRNPU depletion, entirely consistent with the observed decreases in PRC-directed modifications by ChIP-Seq in Figure 4. The RNA-seq data by gene are now reported in Table S5. We are confident in the findings reported and would be surprised if they are not generalizable to other TSC lines and to the embryo in vivo. We will need to secure additional funding to carry out that work particularly in an in vivo context. As they stand, in our opinion, our results are rigorous, interesting, and should inspire deeper investigation. We certainly hope to continue this work in the future.

3) The allele-specific analysis of gene expression at the domains controlled by Airn and Kcnqo1t1, presented in Figures 5A,B, is confusing in that the authors looked at combined effects of target genes. In their earlier studies on this cell line they considered genes individually, and at the Airn domain they showed that some genes had apparent allele-specific gene expression ( Ak029100, Slc22a3 and Mas1). This is not done in the current study and Figure 5A does not provide convincing evidence for allele-specific expression changes at the Airn-controlled locus. The change in the HNRNPU-deficient cells is extremely small in amplitude, with no clear allelism in the WT cells, which in this reviewer’s view prevents conclusions to be drawn about the role of HNRNPU in gene regulation (as is being done in the Abstract and text). It would be helpful if in these analyses the authors would consider individual target genes, particularly the ones that in earlier studies showed clear a allelism in TSCs. They could also consider to differenioate the cells in case this would induce a stronger allelism of the target genes of interest. Possibly, such further studioes would reveal a relevant change in gene expression, or not.

Thank you for this feedback. We have added to the Figure and its accompanying text by including a gene-level analysis of significant changes induced by depletion of HNRNPK and HNRNPU, following an analysis procedure similar to the one we performed to study gene expression after Airn and Kcnq1ot1 truncation in our 2019 study of TSCs (PMID 31256989). We have also renamed this section and the figure title to “HNRNPU and HNRNPK depletions cause low-level derepression of genes in Airn and Xist but not Kcnq1ot1 target domains”, which we feel accurately reflects the reality of our data and perhaps captures the essence of this reviewer’s suggestion.

Consistent with the trends described in our domain-level analyses, we observe many genes in the Airn and Xist target domains whose overall levels of paternal expression increase, albeit by only a small amount, upon depletion of HNRNPK and HNRNPU. The sets of genes whose expression change upon depletion of HNRNPK and HNRNPU are not entirely overlapping; and as the reviewer correctly notes, the levels of derepression are generally low, on the order of 4-8 percent; thus we are underpowered to make informed statements about the possibility of gene-specific mechanisms of repression (page 22 and 23).

Still we have performed in total, 16 biologically replicated RNA-seq experiments to draw these conclusions; by field standards, the experiments are quite rigorous. Regarding interpretations, it may be relevant to note that the perturbations we made are relatively acute (four days of depletion) and are not genetic knockouts, thus had we been able to culture the cells for extended periods of time after protein depletion, and in complete genetic knockouts, we may have observed stronger phenotypes particularly at the level of RNA-seq.

We now report a supplemental table that shows the allelic data analyzed for all genes in the Airn, Kcnq1ot1, and Xist domains across all RNA-seq datasets (Table S5). Reassuringly, the results from these analyses in the (-) dox condition match expectations from our prior works in TSCs in 2012, 2015, 2019, and 2023 (Table S5). Certain genes in the domains are classically imprinted or inactivated genes, while other genes particularly in the Airn target domain, are not classically considered imprinted (and neither do we refer to them as imprinted) but they do exhibit parent of origin expression bias that is sensitive to alterations in Airn expression (PMID 31256989). Consistent with those interpretations, examples from both classes of genes exhibit weak but statistically significant changes in allelic ratios after depletion of HNRNPK and HNRNPU. In the results section, we also now explicitly mention the nine genes in Figure 1 from Schertzer et al. 2019 that were detected with high enough allelic coverage to be included in our current analyses. Of these nine genes, four exhibited significant levels of derepression upon depletion of HNRNPK or HNRNPU or both (*Qk*, *Arid1b*, *Map3k4*, and *Dact2*), and five did not (*Pde10a*, *Slc22a3*, *Igf2r*, *Mas1*, and *Tcp1*). We have taken care in the revised manuscript to talk about the derepression in quantitative but intentionally measured terms. The original abstract did not directly reference the gene expression data but we have expanded the abstract in revision and now briefly mention the data. In the discussion section, we speculate that given their small magnitude, the gene expression changes are secondary effects that arise due to the reductions in PRC-directed modifications (page 29).

4) In case there is indeed a change in the allelism of gene expression in the absence of HNRNPU, it remains unclear whether this is a direct or an indirect effect, and the authors need to be careful in their conclusions. For instance, in Figure 7c it can be seen that Airn lncRNA is less abundant in HNRNPU-deficient cells. This is unexpected given that they also observed a reduced presence of PRC-mediated histone modifications, and may well be an inditect effect of the HNRNPU depletion (the protein presumably controls many factors). Could this reduction in Airn lncRNA have been responsible for the observed minor change in gene allelism in Figure 5A ? This sems a more plausible hypothesis. Further experiments, or considerable rewriting of the conclusions, would be required.

Thank you for sharing this feedback. We note that in our discussion section, we walk through this scenario and state what we believe to be the most likely explanations for our results, which includes the observation that abundance of Airn is reduced by depletion of HNRNPU. At the suggestion of R2, we additionally added a statement about the possibility that HNRNPU is protecting the lncRNAs from degradation by HUSH. We agree that further experimentation is required to fully understand the mutant phenotype and have stated this in the discussion section (pages 29 and 30).

5) The Title wrongly concludes that HNRNPU is required for the recruitment of Polycomb by the lncRNAs Airn and Kcnq1ot1. However, Figure 6 does not show this, and the levels of RING1B binding to the lncRNAs are comparable in the WT and the HNRNPU-deficient cells. In addition, in this context the authors talk about binding of PRC1 to the lncRNAs (eg, last lines of page 22), but can this really be concluded when one studies one PRC1 protein only ? The authors should be more conservative in their conclusions. By the way, the term ‘Polycomb’ is confusing……..does it indicate one protein only, does it indicate ‘Polycomb group’ proteins, or what ?

We respectfully disagree with the reviewer here and think that our differences of opinion may be connected to semantics. In the title, our use of the term recruitment is in reference to effects on chromatin, and in our reading of this comment, we believe the reviewer is perhaps considering that we meant a direct or protein-bridged association between PRC1 and HNRNPU. Regarding which Polycomb complex we are referring to, we and others have shown, including in this work, that PRC1 and PRC2 complexes are recruited in tandem in the regions targeted for repression by Airn, Kcnq1ot1, and Xist. Regarding the study of which PRC1 and PRC2 components interact with Airn, Kcnq1ot1, and Xist, we now directly cite our prior work in TSCs, in which we examined RNA associations with three components of PRC1 and five components of PRC2 (PMID: 40118040). Among the PRC1 and PRC2 components profiled, only RING1B and RYBP exhibited clear associations with Airn, Kcnq1ot1, and Xist, consistent with a model for RNA-mediated PRC recruitment originally proposed by Almeida, Pintacuda, and Brockdorff. At the beginning of the results section in the revised manuscript, we now cite our prior work in a context that more clearly links it to the present study (pages 6 and 7). We have likewise attempted to explain the prior connections between PRC1 and PRC2 more clearly in the revised manuscript. We have changed the title to "Correlated protein-RNA associations and a requirement for HNRNPU in the long-range recruitment of Polycomb Repressive Complexes by the lncRNAs Airn and Kcnq1ot1*"* which is hopefully a bit more specific about our reference to both PRC1 and PRC2. We also added additional language in the abstract to indicate that both PRC1- and PRC2-catalyzed modifications are affected by knockdown of HNRNPU. We avoid the use of the term “binding” throughout the manuscript because the data suggest that neither PRC1 nor PRC2 directly bind Airn, Kcnq1ot1, or Xist (see also PMID 40502125).

6) Presumably, HNRNPU associates with many RNAs throughout the nucleus, in specific and aspecific manners, and thereby influences many processes. In this context, it would be relevant to assess whether HNRNPU is enriched in the vicinity of the Airn and Kcnq1ot1 foci in the nuclei of TSCs. As a conrol, the same could be done for Xist foci. This could be done by combined RNA FISH and IF, similarly as was done by Terranova et al 2008 and by others before. In the presenttaion of RNA FISH studies, it would be helpful to demarcate the nuclei in the shown images. In addition, could ChIP-seq profiles be assessed to ascertain that the protein is associated with chromatin of the target loci (on one parental chromosome mostly) ?

Note that the RIPs we performed using the mouse monoclonal antibody raised against HNRNPU provide evidence that HNRNPU is proximal to Airn, Kcnq1ot1, and Xist in TSCs. The rankings shown in the purple heatmaps of Figure 2A provide evidence to suggest that a greater amount of HNRNPU is associated with Airn, Kcnq1ot1, and Xist than the large majority of other RNAs in the nucleus. The mouse monoclonal antibody raised against FLAG epitope provides additional evidence that HNRNPU is proximal to Xist and Kcnq1ot1 in ESCs. We have in the past attempted ChIPs using the mouse monoclonal antibody raised against HNRNPU and the results did not provide us with any measure of confidence that HNRNPU was interacting with chromatin in a way that would be expected as a DNA-associated factor -- the results looked like pure noise suggesting that HNRNPU is not close enough to chromatin to be crosslinked by ChIP, that it exhibits little specificity in its proximity to chromatin, or that the antibody we used to perform that experiment failed under ChIP crosslinking conditions. We have outlined nuclei in Figure 7 as suggested and believe that the newer version of the figure is improved as a result.

Minor points :

-In the text, the authors mention about used ‘antibodies’ (mo,noclonals ?), nut presumably, in many cases they used antisera. Please, be specific throughout the text. Particularly for polyclonal antosera this is important since they do not represent stable entities.

The reviewer is correct that we used both monoclonal antibodies and polyclonal antisera. We now include a column in Table S6 that displays this information. We have gone through the manuscript to be specific about what entity was used in what section.

-Early in the manuscript, give the full names of ‘CLIP’ and ‘CLAP’ and brkiefly describe what they entail. Not everybody is familiar with these terms.

These terms are defined in the introduction on page 5 and with extra space in the abstract, we have now defined them there, as well.

-It is curious that in the presented studies, SPEN interacts with the 5’ extremities of the analysed lncRNAs. Could the authors comment on what this means, and how this apparent specificity comes about ?

SPEN is a very interesting protein to us. Certainly within Xist and we presume also in Airn and Kcnq1ot1, we believe the major mechanism of its recruitment is driven more by protein-protein interactions than by direct RNA-protein specificity -- see PMID 40502125, which is under review elsewhere. Because SPEN did not become a focus of this study (we attempted to deplete it by CRISPR but were unable to successfully perform western blots on the endogenous protein), we elected to not include this speculation here.
