# Supplementary material for: Correlated protein-RNA associations and a requirement for HNRNPU in the long-range recruitment of Polycomb Repressive Complexes by the lncRNAs Airn and Kcnq1ot1
Source: PLoS Genet. 2026 Jun 23;22(6):e1012215. doi: 10.1371/journal.pgen.1012215 (PMC13318007; doi:10.1371/journal.pgen.1012215)
Supplement: Peer review history

# Peer review: Decision Letter 0

**DOI:** 10.1371/journal.pgen.1012215.r001

**Authors:** John Greally, Michael Blower

11 Apr 2026

PGENETICS-D-26-00217

Correlated protein-RNA associations and a requirement for HNRNPU in long-range Polycomb recruitment by the lncRNAs Airn and Kcnq1ot1

PLOS Genetics

Dear Dr. Calabrese,

Thank you for submitting your manuscript to PLOS Genetics. After careful consideration, we feel that it has merit but does not fully meet PLOS Genetics's publication criteria as it currently stands. Therefore, we invite you to submit a revised version of the manuscript that addresses the points raised during the review process.

Please submit your revised manuscript by Jun 10 2026 11:59PM. If you will need more time than this to complete your revisions, please reply to this message or contact the journal office at plosgenetics@plos.org. When you're ready to submit your revision, log on to https://www.editorialmanager.com/pgenetics/ and select the 'Submissions Needing Revision' folder to locate your manuscript file.

Please include the following items when submitting your revised manuscript:

* A letter that responds to each point raised by the editor and reviewer(s). You should upload this letter as a separate file labeled 'Response to Reviewers'. This file does not need to include responses to any formatting updates and technical items listed in the 'Journal Requirements' section below.

* A marked-up copy of your manuscript that highlights changes made to the original version. You should upload this as a separate file labeled 'Revised Manuscript with Track Changes'.

* An unmarked version of your revised paper without tracked changes. You should upload this as a separate file labeled 'Manuscript'.

If you would like to make changes to your financial disclosure, competing interests statement, or data availability statement, please make these updates within the submission form at the time of resubmission. Guidelines for resubmitting your figure files are available below the reviewer comments at the end of this letter.

We look forward to receiving your revised manuscript.

Kind regards,

Michael D. Blower, Ph.D.

Guest Editor

PLOS Genetics

John Greally

Section Editor

PLOS Genetics

Aimée Dudley

Editor-in-Chief

PLOS Genetics

Anne Goriely

Editor-in-Chief

PLOS Genetics

**Additional Editor Comments:**

In my reading of the manuscript the most important concerns to address are: Reviewer 1 points 3,4,6,7 and 8; Reviewer 2 points 2,3, and 6. Reviewer 2 also raises and important point on p10 of your manuscript that is closely related to the points raised by Reviewer 1. Reviewer 3 points 1 and 3.

**Journal Requirements:**

1) Please ensure that the CRediT author contributions listed for every co-author are completed accurately and in full.

At this stage, the following Authors/Authors require contributions: McKenzie M. Murvin, Shuang Li, Elizabeth W. Abrash, Bridget A. Peck, Samuel P. Boyson, Zhiyue Zhang, Rachel E. Cherney, and Joseph Mauro Calabrese. Please ensure that the full contributions of each author are acknowledged in the "Add/Edit/Remove Authors" section of our submission form.

The list of CRediT author contributions may be found here: https://journals.plos.org/plosgenetics/s/authorship#loc-author-contributions

2) Please upload all main figures as separate Figure files in .tif or .eps format. For more information about how to convert and format your figure files please see our guidelines:

https://journals.plos.org/plosgenetics/s/figures

3) We have noticed that you have uploaded Supporting Information files, but you have not included a list of legends. Please add a full list of legends for your Supporting Information files after the references list.

**Reviewers' comments:**

Reviewer's Responses to Questions

**Comments to the Authors:**

**Please note here if the review is uploaded as an attachment.**

Reviewer #1: In their manuscript, Calabrese and colleagues examine similarities and differences in the networks of lncRNA-protein associations for three previously characterized lncRNAs (Xist, Airn, and Kcnq1ot1), and use these patterns to infer functional and mechanistic features.

The key significance of this manuscript is its focus on a network of 27 RNA binding proteins rather than on individual lncRNA-protein interactions. For this they use a new RIP (crosslinked RNA immunoprecipitation) protocol with validated performance and develop novel computational methods that identify patterns and develop predictive parameters for RNAs with similar/distinct protein association profiles. One of their key findings is that Airn and Kcnq1ot1, two lncRNAs involved in the repression of imprinted regions, have highly similar profiles that are also similar but to a lesser degree to Xist, another repressive lncRNA that acts on a chromosome-scale. Overall, both Xist and imprinting lncRNAs are significantly distinct from most other lncRNAs. The specific distinctions between Airn/Kcnq1ot1 and Xist and between the two imprinted lncRNAs are also highlighted as these might be representative of functional and mechanistic differences. Overall, the first part of the manuscript introduces a new framework for the comprehensive characterization of RNA/protein networks. This work extends prior work by this team that focused on lncRNA sequence kmers to identify functional similarities/differences.

The second part of the manuscript uses information from protein-association networks to select factors that are enriched for all three lncRNAs (hnRNP-K, -U, and Xrn2) or specific to the imprinting lncRNAs (Ythdc1) with predicted or known roles in gene repression. The authors achieve reasonable CRISPR inducible depletion for all 4 factors and perform allele-specific ChIPseq for Polycomb repressive marks (H2AK119Ub and H3K27me3) and gene expression analysis. They observe interesting similarities and differences in how hnRNP-U and hnRNP-K contribute to gene regulation by the three lncRNAs, which the authors interpret as analogous but partly distinct mechanisms of gene repression by the lncRNAs.

Overall, this manuscript lays the foundation for a new network-level approach for addressing the complex and combinatorial nature of lncRNA-protein interactions. The approaches are technically and computationally validated but may need more detailed descriptions in results and figure legends. The visualization of the data is excellent, but labels need to be updated for consistency and clarity. Lastly, jargon related to imprinting and X inactivation fields need more detailed explanations. Overall, improvements in the clarity of text and figures will make this manuscript accessible and of great interest to a wide audience.

(1) Explanation for the use of the specific model system. What are TSCs and why using these cells is appropriate for studying imprinting/X-inactivation?

(2) It is unclear how do the authors define “chromatin-enriched RNAs”?

(3) Figure legends need improvements. In addition to obvious details such as definition of abbreviations (ie MoR and ERCC), panel content should contain descriptions accessible to a wider readership as currently they are too brief and schematic. There should be consistency across figures (ie sgRNA name/number/gene in Fig. 4, 5, and 6 or -/+ dox vs WT/HNRNPU in Fig. 6). Deviations from standard practice should be addressed (ie scale of Read density profiles is usually normalized to allow comparison of peak heights). Fig. 3B there is inconsistency whether 25- or 50-nt bins were used.

(4) Related to (3), the text in Results section is often hard to follow, especially when referring to the methods development section. This is, in part, due to lack of consistent terminology. As one of several examples, Fig 2A is described as representing “summed background-corrected peaks across length,” “overall levels of association,” “RIP signal-under-peak rankings” and “signal-under-peak ranks.” This can be remedied by defining one metric and using it consistently.

(5) Fig. 2C and 3C should also plot the ranking for a few other known lncRNAs that might be of interest readers (such as Malat1, Neat1, Hotair, etc).

(6) There is a disconnect between order of panels in Figs 4 and S6-8 and related text, which makes this section very challenging to understand. The justification for selection of these 4 factors for further analysis based on relationship to PRC1 in Fig. 3A is also not clear.

(7) Jargon in text and figures need to be updated to be more accessible to wider readership – for example when describing maternal/paternal alleles for imprinted loci or discussing degrees of X-inactivation escapees.

(8) Gene expression representation in Figure 5 is challenging to understand. “% paternal” or “% paternal expression bias” needs to be clarified. Best if represented in different colors (B6/Cast -/+dox) across the locus, analogous to Fig. 4. The use of t-test to look across different genes does not seem justified as each gene may be regulated by a distinct mechanism.

(9) In Fig. 7, location of qPCR primers should be indicated.

Reviewer #2: The manuscript entitled "Correlated protein-RNA associations and a requirement for HNRNPU in long-range Polycomb recruitment by the lncRNAs Airn and Kcnq1ot" by Murvin et al. presents a formaldehyde crosslink based method for RNA protein interactions and applies it to investigate the binding of 25 RNA binding factors and 2 Polycomb group proteins to transcripts in B6/CAST hydrid mouse tophectodermal stem cell cultures. The use of hybrid cells allows the allele specific assignments of interactions which is important for detailed analysis of the interactome of the imprinted and non-protein-coding Airn and Kcnq1ot1 RNAs. In addition, crosslinking allows to capture indirect associated proteins, which is used to determine the dependence of interactions on the RNA binding protein hnRNPU, which is identified as an interactor of Airn, Kcnq1ot1, and Xist.

Comparison between the 3 lncRNAs based on extended analysis using available databases of protein interactions indicates similarities and differences, whose functional significance remains untested. The authors further observe a role for HNRNPU for establishment of the Polycomb complex associated histone modifications H3K27ME3 and H2AUB by performing sgRNA mediated depletion. This is an interesting observation as hnRNPU has not been directly implicated in Polycomb recruitment before, but in the localization of Xist RNA to the inactive X chromosome. A similar conclusion for AIRN and KCNQ1OT1 cannot be made suggesting potentially divergent requirements for hnRNPU between different non-coding RNAs.

The study can make an advance on the methodology for RNA-protein interactions, although the present version falls short in clearly positioning the new method over earlier publications using crosslinking and antibody mediated enrichment strategies. The biological insight gained is limited but might be a valuable resource for protein binding in mouse trophoblast stem cells, which could be of interest for researchers in RNA biology and stem cells. Before publication can be considered a number of points should be addressed by the authors to improve the clarity and strengthen the conclusions of the study:

Specific points

1. The authors use 6 replicates with non-targeting IgG to control for specificity. However, this does not rule out any cross-reactivity of the actual antisera used for the different interactors. This is a critical issue as the interpretation and usefulness of the binding data as a resource depends on. Therefore, controls for antibody specificity in addition to IgG are needed. The data in Fig. S2 Western blot raises this concern directly as multiple bands for ALYREF, LBR, TIA1, and NXF1 are observed. In addition, the antisera for detecting CIZ is stated to only yield specific bands by IP WB, which in the figure panel also does not appear as a single clear band. The image for MATR3 MW seems to indicate 125 and 95 KDa expected, but the band seems to be closer to the number 50, which makes it unclear how to interpret this critical control.

2. The conclusion at the end of the section on page 12 "Thus, the protein association profiles measured over Airn and Kcnq1ot1 are more similar to each other than to the vast majority of other chromatin-enriched RNAs and exhibit a more moderate degree of similarity to Xist." raises the question if the differences would be explained alone by the greater abundance of Xist relative to Airn/Kcnq1ot1. It could also be considered that the half-life of these RNAs when let us say the RNA binding proteins were grouped by their presumed on rates. As the section seems to contain a large number of statistical detail including rank numbers, the overall positioning of what this can tell us should be improved to make a convincing test case for the usefulness of the method.

2. In Figure 3 the peaks for Ciz1 and PTBP1 seem to overlap with peaks at the same positions the in IgG control. Maybe I have misunderstood the figure, but an explanation is needed as if indeed the peaks would be observed in the IgG control how could this experiment be interpreted?

3. I am wondering if the sharp peaks which the authors observe for Rybp and Ring1b might be caused by DNA contamination? In this case it is conceivable that the proteins are binding chromatin independent of RNA and a clarification to what extent contamination with chromatin DNA can be ruled out should be added.

4. Tsix overlaps Xist on the reverse strand and has also been implicated as a major repressive RNA. It would be interesting to know if the authors have detected Tsix in their FLAG-HNRNPK and FLAG-HNRNPU RIP discussed in the beginning of the results section. As the data is likely available, this could be an interesting case as Tsix is published as expressed in mouse ES cells and might be of a similar abundance as Airn or Kcnq1ot1.

5. Fig. S8 shows that the depletion of XRN and YTHDC1 is reducing but likely not completely eliminating the protein leaving the possibility open that these proteins might require a lower abundance for fulfilling their functions. Accordingly this limitation should be added to the text on page 17 line4 when concluding that both proteins are not essential.

6. On the lower part of page 20 the authors explain hat their protocol has been adapted from an earlier fRIP method which has been used by the authors before. On one hand this seems to serve to reassure that the method can provide high quality data, but on the other hand this statement also leaves the novelty of the method unclear. The positioning of the study would be much improved by clearly discussing the innovation in this manuscript against earlier work. This is important as the biological advances that can be made appear rather limited, and a clear advance on the methodology could strengthen the impact of the study.

Minor points

a) Pg 6: "non-FLAG antibodies" could be replaced by a more specific expression

b) Pg 10: "CLAP generally although not always recovers less noise, whereas RIP can recover higher signal intensity under peak regions, presumably due to efficiency of formaldehyde crosslinking": I am wondering if the conclusion on page 10 could be rephrased to indicate specificity and non-specific binder as better quantified either by probabilistic modelling of absolute percentages. Noise and signal seem to be meaningful only in the computational domain but it is difficult to understand a molecular biochemical relation. It could be considered to replace noise, background and other synonyms with non specific binders throughout the text to easy understanding, unless their is a specific reason for the synonym which should be made clear to the reader.

c) Unspliced lncRNA have recently been proposed as being targets of the HUSH complex. The authors emphasize a role of Polycomb but does not consider HUSH contributions to gene silencing.Since all 3 RNAs have large first exons and likely limited splicing a reference to the literature and why HUSH dependence on HNRNPU or K has not been considered should be included in the discussion.

d) The references for the requirements of HNRNPK and SPEN for silencing should be separated on page 4 last sentence to make attribution clearer.

Reviewer #3: In this thorough, well presented study by the team of Mauro Calabrese, the authors explored which proteins could be associated with the regulatory lncRNAs Xist, Kcnq1ot1 and Airn in murine TSCs. As a basis, they took all the proteins known to be associated with Xist. Importantly, they compared a novel, adapted RIP protocol with existing CLIP and CLAP methodologies previously published by others. This allowed them to determine with a higher degree of certainty which proteins are associated with the three different lncRNAs. Interestingly, they find patterns of protein associations that are similar between Airn and Kcnq1ot1, and one of the new proteins identified was HNRNP-U. In a second part of the study, they performed functional studies on this protein and found that its association is important for the maintenance of histone methylation controlled by PRC1 and PRC2 complexes. In addition, they describe an effect of this protein on the allelic repression of target genes of Airn, but here the data are less convincing. Overall, this study provides novel insights into the gene repression controlled by the three lncRNA, and represents an important to the field. Nevertheless, this reviewer has several major concerns and about the data and their interpretation, and this is explained below.

Major concerns :

1) The conclusions are mostly based on analysis of a single mouse hybrid TSC line only. This line was derived many years ago (Calabrese et al. 2012). Since stem cells can readily deviate during in vitro culture, and upon manipulation, both epigenetically and genetically, it is important to ensure that this single female TSC line was still appropriate for the studoies at the analysed passages. In particular, the authors need to demonstrate that the allele-specific DNA methylation at the promoter regions of Airn and Kcnq1ot1 is unaltered in these female TSCs at the passages used (maternal allele only, 50% overall level). To be able to draw general conclusions, it is also important to demonstrate that this cell line had an unaltered karyotype.

2) Ideally, however, the authors should complement their data with a selective experiments on an independent second TSC line, if possible of reciprocal genotype. Such lelective experiments on a second line could confirm their finding that HNRNPU influences levels of allele-specific histone modifications controlled by PRC complexes, and could strengthen the supposed effects on the levels and allele-specificity of expression of the Airn target genes.

3) The allele-specific analysis of gene expression at the domains controlled by Airn and Kcnqo1t1, presented in Figures 5A,B, is confusing in that the authors looked at combined effects of target genes. In their earlier studies on this cell line they considered genes individually, and at the Airn domain they showed that some genes had apparent allele-specific gene expression ( Ak029100, Slc22a3 and Mas1). This is not done in the current study and Figure 5A does not provide convincing evidence for allele-specific expression changes at the Airn-controlled locus. The change in the HNRNPU-deficient cells is extremely small in amplitude, with no clear allelism in the WT cells, which in this reviewer’s view prevents conclusions to be drawn about the role of HNRNPU in gene regulation (as is being done in the Abstract and text). It would be helpful if in these analyses the authors would consider individual target genes, particularly the ones that in earlier studies showed clear a allelism in TSCs. They could also consider to differenioate the cells in case this would induce a stronger allelism of the target genes of interest. Possibly, such further studioes would reveal a relevant change in gene expression, or not.

4) In case there is indeed a change in the allelism of gene expression in the absence of HNRNPU, it remains unclear whether this is a direct or an indirect effect, and the authors need to be careful in their conclusions. For instance, in Figure 7c it can be seen that Airn lncRNA is less abundant in HNRNPU-deficient cells. This is unexpected given that they also observed a reduced presence of PRC-mediated histone modifications, and may well be an inditect effect of the HNRNPU depletion (the protein presumably controls many factors). Could this reduction in Airn lncRNA have been responsible for the observed minor change in gene allelism in Figure 5A ? This sems a more plausible hypothesis. Further experiments, or considerable rewriting of the conclusions, would be required.

5) The Title wrongly concludes that HNRNPU is required for the recruitment of Polycomb by the lncRNAs Airn and Kcnq1ot1. However, Figure 6 does not show this, and the levels of RING1B binding to the lncRNAs are comparable in the WT and the HNRNPU-deficient cells. In addition, in this context the authors talk about binding of PRC1 to the lncRNAs (eg, last lines of page 22), but can this really be concluded when one studies one PRC1 protein only ? The authors should be more conservative in their conclusions. By the way, the term ‘Polycomb’ is confusing……..does it indicate one protein only, does it indicate ‘Polycomb group’ proteins, or what ?

6) Presumably, HNRNPU associates with many RNAs throughout the nucleus, in specific and aspecific manners, and thereby influences many processes. In this context, it would be relevant to assess whether HNRNPU is enriched in the vicinity of the Airn and Kcnq1ot1 foci in the nuclei of TSCs. As a conrol, the same could be done for Xist foci. This could be done by combined RNA FISH and IF, similarly as was done by Terranova et al 2008 and by others before. In the presenttaion of RNA FISH studies, it would be helpful to demarcate the nuclei in the shown images. In addition, could ChIP-seq profiles be assessed to ascertain that the protein is associated with chromatin of the target loci (on one parental chromosome mostly) ?

Minor points :

-In the text, the authors mention about used ‘antibodies’ (mo,noclonals ?), nut presumably, in many cases they used antisera. Please, be specific throughout the text. Particularly for polyclonal antosera this is important since they do not represent stable entities.

-Early in the manuscript, give the full names of ‘CLIP’ and ‘CLAP’ and brkiefly describe what they entail. Not everybody is familiar with these terms.

-It is curious that in the presented studies, SPEN interacts with the 5’ extremities of the analysed lncRNAs. Could the authors comment on what this means, and how this apparent specificity comes about ?

**********

**Have all data underlying the figures and results presented in the manuscript been provided?**

Large-scale datasets should be made available via a public repository as described in the *PLOS Genetics*  data availability policy, and numerical data that underlies graphs or summary statistics should be provided in spreadsheet form as supporting information.

Reviewer #1: Yes

Reviewer #2: None

Reviewer #3: Yes

**********

PLOS authors have the option to publish the peer review history of their article (what does this mean?). If published, this will include your full peer review and any attached files.

If you choose “no”, your identity will remain anonymous but your review may still be made public.

**Do you want your identity to be public for this peer review?**  For information about this choice, including consent withdrawal, please see our Privacy Policy.

Reviewer #1: No

Reviewer #2: No

Reviewer #3: **Yes:** Robert Feil

[NOTE: If reviewer comments were submitted as an attachment file, they will be attached to this email and accessible via the submission site. Please log into your account, locate the manuscript record, and check for the action link "View Attachments". If this link does not appear, there are no attachment files.]

**Figure resubmission:**

-->While revising your submission, we strongly recommend that you use PLOS’s NAAS tool (https://ngplosjournals.pagemajik.ai/artanalysis) to test your figure files. NAAS can convert your figure files to the TIFF file type and meet basic requirements (such as print size, resolution), or provide you with a report on issues that do not meet our requirements and that NAAS cannot fix.

After uploading your figures to PLOS’s NAAS tool - https://ngplosjournals.pagemajik.ai/artanalysis, NAAS will process the files provided and display the results in the "Uploaded Files" section of the page as the processing is complete. If the uploaded figures meet our requirements (or NAAS is able to fix the files to meet our requirements), the figure will be marked as "fixed" above. If NAAS is unable to fix the files, a red "failed" label will appear above. When NAAS has confirmed that the figure files meet our requirements, please download the file via the download option, and include these NAAS processed figure files when submitting your revised manuscript.

**Reproducibility:**

To enhance the reproducibility of your results, we recommend that authors of applicable studies deposit laboratory protocols in protocols.io, where a protocol can be assigned its own identifier (DOI) such that it can be cited independently in the future. Additionally, PLOS ONE offers an option to publish peer-reviewed clinical study protocols. Read more information on sharing protocols at https://plos.org/protocols?utm_medium=editorial-email&utm_source=authorletters&utm_campaign=protocols

# Author comment: Author response to Decision Letter 1

**DOI:** 10.1371/journal.pgen.1012215.r002

20 May 2026

Attachment Submitted filename: plos_G_reviews_v8.docx

# Peer review: Decision Letter 1

**DOI:** 10.1371/journal.pgen.1012215.r003

**Authors:** John Greally, Michael Blower, John Greally, Michael Blower

10 Jun 2026

PGENETICS-D-26-00217R1

Correlated protein-RNA associations and a requirement for HNRNPU in the long-range recruitment of Polycomb Repressive Complexes by the lncRNAs Airn and Kcnq1ot1

PLOS Genetics

Dear Dr. Calabrese,

Thank you for submitting your manuscript to PLOS Genetics. After careful consideration, we feel that it has merit but does not fully meet PLOS Genetics's publication criteria as it currently stands. Therefore, we invite you to submit a revised version of the manuscript that addresses the points raised during the review process.

Please submit your revised manuscript within by Jul 10 2026 11:59PM. If you will need more time than this to complete your revisions, please reply to this message or contact the journal office at plosgenetics@plos.org. When you're ready to submit your revision, log on to https://www.editorialmanager.com/pgenetics/ and select the 'Submissions Needing Revision' folder to locate your manuscript file.

Please include the following items when submitting your revised manuscript:

* A letter that responds to each point raised by the editor and reviewer(s). You should upload this letter as a separate file labeled 'Response to Reviewers'. This file does not need to include responses to formatting updates and technical items listed in the 'Journal Requirements' section below.

* A marked-up copy of your manuscript that highlights changes made to the original version. You should upload this as a separate file labeled 'Revised Manuscript with Track Changes'.

* An unmarked version of your revised paper without tracked changes. You should upload this as a separate file labeled 'Manuscript'.

If you would like to make changes to your financial disclosure, competing interests statement, or data availability statement, please make these updates within the submission form at the time of resubmission. Guidelines for resubmitting your figure files are available below the reviewer comments at the end of this letter.

As the corresponding author, your ORCID iD is verified in the submission system and will appear in the published article. PLOS supports the use of ORCID, and we encourage all coauthors to register for an ORCID iD and use it as well. Please encourage your coauthors to verify their ORCID iD within the submission system before final acceptance, as unverified ORCID iDs will not appear in the published article. Only the individual author can complete the verification step; PLOS staff cannot verify ORCID iDs on behalf of authors.

We look forward to receiving your revised manuscript.

Kind regards,

Michael D. Blower, Ph.D.

Guest Editor

PLOS Genetics

John Greally

Section Editor

PLOS Genetics

Aimée Dudley

Editor-in-Chief

PLOS Genetics

Anne Goriely

Editor-in-Chief

PLOS Genetics

**Additional Editor Comments (if provided):**

Dear Dr. Calabrese,

Thank you for submitting your revised manuscript. After review two of the reviewers agree that your manuscript is significantly improved. Reviewer #1 does not support publication because they do not see the value in benchmarking your fRIP method against CLIP and CLAP. However, I agree with you that head-to-head comparisons of RNA:protein interaction methods is an important and timely topic. Reviewer #3 still has a significant concern about your discussion of the mechanism through which HNRNPU depletion leads to derepression of genes silenced by Airn. Reading through your discussion I see that you have addressed this issue, but have not explicitly addressed the possibility that a reduction in Airn (or Xist) expression could lead to derepression of Airn-silenced genes. I think that you can address this point by modifying the sentence on lines 717-719 in your discussion to directly state that derepression of Airn-silenced genes could result from a loss of Airn expression in HNRNPU knockdown cells.

Please also address the additional points raised by Reviewer #3.

Sincerely,

Michael Blower

**Journal Requirements:**

1) Please ensure that the CRediT author contributions listed for every co-author are completed accurately and in full.

At this stage, the following Authors/Authors require contributions: Zhiyue Zhang. Please ensure that the full contributions of each author are acknowledged in the "Add/Edit/Remove Authors" section of our submission form.

The list of CRediT author contributions may be found here: https://journals.plos.org/plosgenetics/s/authorship#loc-author-contributions

2) We have noticed that you have a list of Supporting Information legends in your manuscript. However, there are no corresponding files uploaded to the submission. Please upload them as separate files with the item type 'Supporting Information'.

3) We have amended your Competing Interest statement to comply with journal style. We kindly ask that you double check the statement and let us know if anything is incorrect.

4) Thank you for submitting your data to a repository. We have been unable to access the data using the accession number you provided. Please contact the repository to ensure that the accession numbers are valid. The accession number is still private. Kindly revise the link into public to access data. If a link or accession number needs updating, please provide a revised Data Availability Statement including the information.

-->5)

Your current Financial Disclosure states, "T-->his work was supported by NIH grants R01GM136819, R01GM121806, and R35GM153293; NSF grant DBI-2228805; and the Yang Family Biomedical Scholar Fund (J.M.C.). M.M.M. was supported by T32GM119999 and F31HD111292. R.E.C. was supported by T32GM007092 and F31HD103334. E.W.A was supported by F31HD114456 and a Marzluff Graduate Fellowship from the RNA Discovery Center. The sponsors/funders played no role in the study design, data collection and analysis, decision to publish, or preparation of the manuscript. NIGMS: https://www.nigms.nih.gov, NICHD:, https://www.nichd.nih.gov, NSF DBI:

https://www.nsf.gov/bio/dbi/about, UNC RNA Discovery Center: https://unclineberger.org/rnadiscoverycenter/-->"

However, your funding information on the submission form is missing the funder name "Eunice Kennedy Shriver National Institute of Child Health and Human Development" and "National Institute of General Medical Sciences" and Grant recipient mismatches for grant number "T32GM007092". Please indicate by return email the full and correct funding information for your study and confirm the order in which funding contributions should appear. Please be sure to indicate whether the funders played any role in the study design, data collection and analysis, decision to publish, or preparation of the manuscript.

**Reviewers' comments:**

Reviewer's Responses to Questions

**Comments to the Authors:**

**Please note here if the review is uploaded as an attachment.**

Reviewer #1: The revised version addresses all my concerns. Recommend for publication.

Reviewer #2: The revised version of the manuscript entitled "Correlated protein-RNA associations and a requirement for HNRNPU in the long-range recruitment of Polycomb Repressive Complexes by the lncRNAs Airn and Kcnq1ot1" by Murvin et al., contains clarifications to the text and improved data presentation that together have greatly enhanced the readability and clarity of the narrative and make understanding of the study possible. These changes have also led to a reduction of my initial enthusiasm as it appears after considering the response of the authors to my points earlier I had overestimated the advance and quality at my optimistic initial reading. In particular, the authors now clearly state that there in no novelty of the method and they understand the value of their present study as benchmarking their method against other methods. However, I find this a less impressive positioning as it appears quite frankly unscientific to provide important benchmarking after a method has been extensively used for conclusions in other studies. One would have hoped that the validity of the method be established before conclusions are drawn. Now post-hoc reading a confirmatory evaluation by the authors themselves leaves the sour taste that this can unlikely be without prejudice as their earlier conclusions must have dependent on the - back then unknown - validity of the methodology. Secondly, I an allerted by an, albeit honest, lengthy and extensive limitations section of the discussion. In my opinion some of the conclusions might indeed be on uncertain grounds in particular related to cross reactivity of some antisera. However, mixing robust conclusions with preliminary and likely uncertain once does the authors a great deal of disfavour. I would still think that the results that are corroberated with tagged versions and complementary evidence are of interest. Interlacing these valid conclusions with a large set of unverified speculations based on dubious antisera, confuses the reader, who would have to on a case by case basis dissect the evidence. In my mind the benchmarking on the majority of the antisera panel is suitable for a technical archival journal also in recognition of the need by the authors to establish the methodology sincerely as the rebuttal points out. However, the quality and advance of this is not suitable for a journal of a broad readership as PLoS Genetics. The biological insight on hnRNPU and Polycomb recruitment would best be communicated in a short article. In conclusion, I feel that the revision has certainly clarified the study but not strengthened its impact. In its current form I hesitate that it will be well received and can make an impact in the scientific community.

Reviewer #3: The authors present a carefully rewritten manuscript, with additional information and many changes in the way the data are presented and discussed. The revision improved considerably the manuscript, which is now also more accessible to a broader readership. All but one of the points raised by this reviewer were addressed by the authors.

Remaining major Point :

The authors did not address a main points raised by this reviewer (point 4):

In case there is indeed a change in the allelism of gene expression in the absence of HNRNPU, it remains unclear whether this is a direct or an indirect effect, and the authors need to be careful in their conclusions. For instance, in Figure 7c it can be seen that Airn lncRNA is less abundant in HNRNPU-deficient cells. This is unexpected given that they also observed a reduced presence of PRC-mediated histone modifications, and may well be an inditect effect of the HNRNPU depletion (the protein presumably controls many factors). Could this reduction in Airn lncRNA have been responsible for the observed minor change in gene allelism in Figure 5A? This seems a more plausible hypothesis. Further experiments, or considerable rewriting of the conclusions, would be required.

In the Discussion, the authors have to present and discuss the alternative model, namely that the minor allelic derepression of genes at the Airn-controlled domain in the HNRNPU deficient cells is an indirect consequence of the reduction in Airn levels-(of about 30%, Fig. 7c). A same reasoning could be applied to Xist, whose level is about 40% reduced in the HNRNPU-deficient cells. Given the importance of the dosage of these lncRNAs for their chromatin repressive effects, reported by the authors themselves and others in earlier studies, it is essential to indicate and discuss this plausible alternative hypothesis.

Incidentally, the authors show that the lncRNA Kcnq1ot1 is not reduced in its level in the HNRNPU deficient cells, and at the target locus of this lncRNA, the HNRNPU depletion does not lead to allelic gene repression. Also this finding would agree with an alternative model in which the observed allelic repression changes arise dependent on whether the HNRNPU depletion affects the levels of the lncRNA or not.

Minor points :

-Figure 2A : Should the association of Malat1 to U2AF35 not be indicated in purple (because ranking =1) ?

-Line 541 : FIG ?

**********

**Have all data underlying the figures and results presented in the manuscript been provided?**

Large-scale datasets should be made available via a public repository as described in the *PLOS Genetics*  data availability policy, and numerical data that underlies graphs or summary statistics should be provided in spreadsheet form as supporting information.

Reviewer #1: Yes

Reviewer #2: Yes

Reviewer #3: Yes

**********

PLOS authors have the option to publish the peer review history of their article (what does this mean?). If published, this will include your full peer review and any attached files.

If you choose “no”, your identity will remain anonymous but your review may still be made public.

**Do you want your identity to be public for this peer review?**  For information about this choice, including consent withdrawal, please see our Privacy Policy.

Reviewer #1: No

Reviewer #2: No

Reviewer #3: **Yes:** Robert Feil

[NOTE: If reviewer comments were submitted as an attachment file, they will be attached to this email and accessible via the submission site. Please log into your account, locate the manuscript record, and check for the action link "View Attachments". If this link does not appear, there are no attachment files.]

**Figure resubmission:**

-->While revising your submission, we strongly recommend that you use PLOS’s NAAS tool (https://ngplosjournals.pagemajik.ai/artanalysis) to test your figure files. NAAS can convert your figure files to the TIFF file type and meet basic requirements (such as print size, resolution), or provide you with a report on issues that do not meet our requirements and that NAAS cannot fix.

After uploading your figures to PLOS’s NAAS tool - https://ngplosjournals.pagemajik.ai/artanalysis, NAAS will process the files provided and display the results in the "Uploaded Files" section of the page as the processing is complete. If the uploaded figures meet our requirements (or NAAS is able to fix the files to meet our requirements), the figure will be marked as "fixed" above. If NAAS is unable to fix the files, a red "failed" label will appear above. When NAAS has confirmed that the figure files meet our requirements, please download the file via the download option, and include these NAAS processed figure files when submitting your revised manuscript.

**Reproducibility:**

To enhance the reproducibility of your results, we recommend that authors deposit laboratory protocols in protocols.io, where a protocol can be assigned its own identifier (DOI) such that it can be cited independently in the future. Additionally, PLOS ONE offers an option to publish peer-reviewed clinical study protocols. Read more information on sharing protocols at https://plos.org/protocols?utm_medium=editorial-email&utm_source=authorletters&utm_campaign=protocols

# Author comment: Author response to Decision Letter 2

**DOI:** 10.1371/journal.pgen.1012215.r004

15 Jun 2026

Attachment Submitted filename: response_to_reviews_round2_v1.docx

# Editor report: Decision Letter 2

**DOI:** 10.1371/journal.pgen.1012215.r005

**Authors:** John Greally, Michael Blower, John Greally, Michael Blower, John Greally, Michael Blower

16 Jun 2026

Dear Dr Calabrese,

We are pleased to inform you that your manuscript entitled "Correlated protein-RNA associations and a requirement for HNRNPU in the long-range recruitment of Polycomb Repressive Complexes by the lncRNAs Airn and Kcnq1ot1" has been editorially accepted for publication in PLOS Genetics. Congratulations!

Before your submission can be formally accepted and sent to production you will need to complete our formatting changes, which you will receive in a follow up email. Please be aware that it may take several days for you to receive this email; during this time no action is required by you. Please note: the accept date on your published article will reflect the date of this provisional acceptance, but your manuscript will not be scheduled for publication until the required changes have been made.

Once your paper is formally accepted, an uncorrected proof of your manuscript will be published online ahead of the final version, unless you’ve already opted out via the online submission form. If, for any reason, you do not want an earlier version of your manuscript published online or are unsure if you have already indicated as such, please let the journal staff know immediately at plosgenetics@plos.org.

In the meantime, please log into Editorial Manager at https://www.editorialmanager.com/pgenetics/, click the "Update My Information" link at the top of the page, and update your user information to ensure an efficient production and billing process. Note that PLOS requires an ORCID iD for all corresponding authors. Therefore, please ensure that you have an ORCID iD and that it is validated in Editorial Manager. To do this, go to ‘Update my Information’ (in the upper left-hand corner of the main menu), and click on the Fetch/Validate link next to the ORCID field.  This will take you to the ORCID site and allow you to create a new iD or authenticate a pre-existing iD in Editorial Manager.

If you have a press-related query, or would like to know about making your underlying data available (as you will be aware, this is required for publication), please see the end of this email. If your institution or institutions have a press office, please notify them about your upcoming article at this point, to enable them to help maximise its impact. Inform journal staff as soon as possible if you are preparing a press release for your article and need a publication date.

Thank you again for supporting open-access publishing; we are looking forward to publishing your work in PLOS Genetics!

Yours sincerely,

Michael D. Blower, Ph.D.

Guest Editor

PLOS Genetics

John Greally

Section Editor

PLOS Genetics

Aimée Dudley

Editor-in-Chief

PLOS Genetics

Anne Goriely

Editor-in-Chief

PLOS Genetics

www.plosgenetics.org

BlueSky: @plos.bsky.social

----------------------------------------------------

Comments from the reviewers (if applicable):

----------------------------------------------------

**Data Deposition**

If you have submitted a Research Article or Front Matter that has associated data that are not suitable for deposition in a subject-specific public repository (such as GenBank or ArrayExpress), one way to make that data available is to deposit it in the Dryad Digital Repository. As you may recall, we ask all authors to agree to make data available; this is one way to achieve that. A full list of recommended repositories can be found on our website.

The following link will take you to the Dryad record for your article, so you won't have to re‐enter its bibliographic information, and can upload your files directly:

http://datadryad.org/submit?journalID=pgenetics&manu=PGENETICS-D-26-00217R2

More information about depositing data in Dryad is available at http://www.datadryad.org/depositing. If you experience any difficulties in submitting your data, please contact help@datadryad.org for support.

Additionally, please be aware that our data availability policy requires that all numerical data underlying display items are included with the submission, and you will need to provide this before we can formally accept your manuscript, if not already present.

----------------------------------------------------

**Press Queries**

If you or your institution will be preparing press materials for this manuscript, or if you need to know your paper's publication date for media purposes, please inform the journal staff as soon as possible so that your submission can be scheduled accordingly. Your manuscript will remain under a strict press embargo until the publication date and time. This means an early version of your manuscript will not be published ahead of your final version. PLOS Genetics may also choose to issue a press release for your article. If there's anything the journal should know or you'd like more information, please get in touch via plosgenetics@plos.org.

# Editor report: Acceptance letter

**DOI:** 10.1371/journal.pgen.1012215.r006

**Authors:** John Greally, Michael Blower, John Greally, Michael Blower, John Greally, Michael Blower

PGENETICS-D-26-00217R2

Correlated protein-RNA associations and a requirement for HNRNPU in the long-range recruitment of Polycomb Repressive Complexes by the lncRNAs Airn and Kcnq1ot1

Dear Dr Calabrese,

We are pleased to inform you that your manuscript entitled "Correlated protein-RNA associations and a requirement for HNRNPU in the long-range recruitment of Polycomb Repressive Complexes by the lncRNAs Airn and Kcnq1ot1" has been formally accepted for publication in PLOS Genetics! Your manuscript is now with our production department and you will be notified of the publication date in due course.

The corresponding author will soon be receiving a typeset proof for review, to ensure errors have not been introduced during production. Please review the PDF proof of your manuscript carefully, as this is the last chance to correct any errors. Please note that major changes, or those which affect the scientific understanding of the work, will likely cause delays to the publication date of your manuscript.

Soon after your final files are uploaded, unless you have opted out or your manuscript is a front-matter piece, the early version of your manuscript will be published online. The date of the early version will be your article's publication date. The final article will be published to the same URL, and all versions of the paper will be accessible to readers.

For Research Articles, you will receive an invoice from PLOS for your publication fee after your manuscript has reached the completed accept phase. If you receive an email requesting payment before acceptance or for any other service, this may be a phishing scheme. Learn how to identify phishing emails and protect your accounts at https://explore.plos.org/phishing.

Thank you again for supporting PLOS Genetics and open-access publishing. We are looking forward to publishing your work!

With kind regards,

Sharmila Kamatchi

PLOS Genetics

On behalf of:

The PLOS Genetics Team

Carlyle House, Carlyle Road, Cambridge CB4 3DN | United Kingdom

plosgenetics@plos.org | +44 (0) 1223-442823

plosgenetics.org | Twitter: @PLOSGenetics
